# The prognostic role of ACSL4 in postoperative adjuvant TACE-treated HCC: implications for therapeutic response and mechanistic insights

**DOI:** 10.1186/s13046-024-03222-5

**Published:** 2024-11-19

**Authors:** Ji Feng, Jin-Lian Bin, Xi-Wen Liao, Yong Wu, Yue Tang, Pei-Zhi Lu, Guang-Zhi Zhu, Qian-Ru Cui, Yock Young Dan, Guo-Huan Yang, Li-Xin Li, Jing-Huan Deng, Tao Peng, Shing Chuan Hooi, Jing Zhou, Guo-Dong Lu

**Affiliations:** 1 School of Public Health, Fudan University, 130 Dong-An Road, Shanghai, 200032 China; 2 Department of Toxicology, School of Public Health, Guangxi Medical University, Nanning, Guangxi 530021 China; 3 Key Laboratory of Basic Research On Regional Diseases (Guangxi Medical University), Education Department of Guangxi Zhuang Autonomous Region, Nanning, Guangxi 530021 China; 4 Department of Hepatobiliary Surgery, The First Affiliated Hospital of Guangxi Medical University, 6 Shuangyong Road, Nanning, Guangxi 530021 China; 5 Department of Physiology, School of Preclinical Medicine, Guangxi Medical University, 22 Shuangyong Road, Nanning, Guangxi 530021 China; 6 Department of Medicine, Yong Loo Lin School of Medicine, National University of Singapore, Singapore, 119228 Singapore; 7 Department of Liver Surgery and Transplantation, Zhongshan Hospital, Fudan University, Shanghai, 200032 China; 8 Department of Hepato-Oncology, Zhongshan Hospital, Fudan University, Shanghai, 200032 China; 9 Key Laboratory of Early Prevention and Treatment for Regional High Frequency Tumor (Guangxi Medical University), Ministry of Education; Guangxi Key Laboratory of High-Incidence-Tumor Prevention & Treatment, (Guangxi Medical University), Nanning, Guangxi 530021 China; 10 Department of Physiology, Yong Loo Lin School of Medicine, National University of Singapore, 2 Medical Drive MD9, Singapore, 117593 Singapore

**Keywords:** HCC, TACE, ACSL4, Glucose starvation, Canagliflozin

## Abstract

**Background:**

The response of hepatocellular carcinoma (HCC) to transarterial chemoembolization (TACE) treatment and its underlying mechanisms remain elusive. This study investigates the role of enzymes involved in fatty acid activation, specifically Acyl-CoA synthetase long chain 4 (ACSL4), in HCC patients treated with postoperative adjuvant TACE (PA-TACE) and in nutrient-deprived HCC cells.

**Methods:**

We examined the expression of ACSL4 and its family members in HCC clinical samples and cell lines. The clinical significance of ACSL4, particularly regarding the prognosis of patients treated with PA-TACE, was assessed using two independent HCC cohorts. We further explored the role of ACSL4 in glucose starvation-induced cell death in HCC cells and xenograft mouse models.

**Results:**

Among the family members, ACSL4 is the most up-regulated enzyme, associated with poor survival in HCC patients, particularly in post-recurrent TACE-treated patients in a Singapore cohort. ACSL4 is essential for HCC cell survival in response to glucose starvation, rather than to hypoxia or to the combination of hypoxia with doxorubicin or cisplatin. ACSL4-mediated arachidonic acid (AA) metabolism supports mitochondrial *β*-oxidation and energy production. CCAAT/enhancer binding protein α (CEBPA) transcriptionally regulates ACSL4 by binding 3 motifs (-623 to -613, -1197 to -1187 and -1745 to -1735) of ACSL4 upstream promoter region, enhancing its pro-survival effects. Furthermore, canagliflozin (Cana), a clinical-approved drug for type 2 diabetes, mimics glucose starvation and inhibits the growth of ACSL4-low xenograft tumors. Moreover, high ACSL4 or CEBPA expressions correlate with increased recurrence susceptibility after PA-TACE in the China-Guangxi HCC cohort.

**Conclusions:**

The CEBPA-ACSL4 pathway is critical in protecting HCC cells from glucose starvation-induced cell death, suggesting that ACSL4 and CEBPA could serve as valuable prognostic indicators and potential therapeutic targets in the context of PA-TACE treatment for HCC.

**Graphical Abstract:**

TACE is the first-line treatment for intermediate-stage HCC patients with unresectable tumor and a common postoperative adjuvant (PA) treatment. The present study found that HCC patients with the presence of CEBPA-ACSL4 expression in tumor are more resistant to TACE, susceptible for PA-TACE relapse and poorer survival. Mechanically, ACSL4 is essential for fatty acid activation particularly conversion of arachidonic acid (AA) into AA-CoA, which promotes lipid anabolism in nutrition-replete condition and lipid catabolism in glucose-deplete condition. As a result, ACSL4-high HCC cells, in response to glucose restriction (rather than hypoxia and chemotherapeutic drugs), can donor mitochondrial energy production through *β*-oxidation and protect HCC cells from mitochondrial membrane impairment and cell death *in vitro* and *in vivo*. In addition, CEBPA transcriptionally activates ACSL4 , and knockout of CEBPA aborted ACSL4-mediated lipid metabolism.

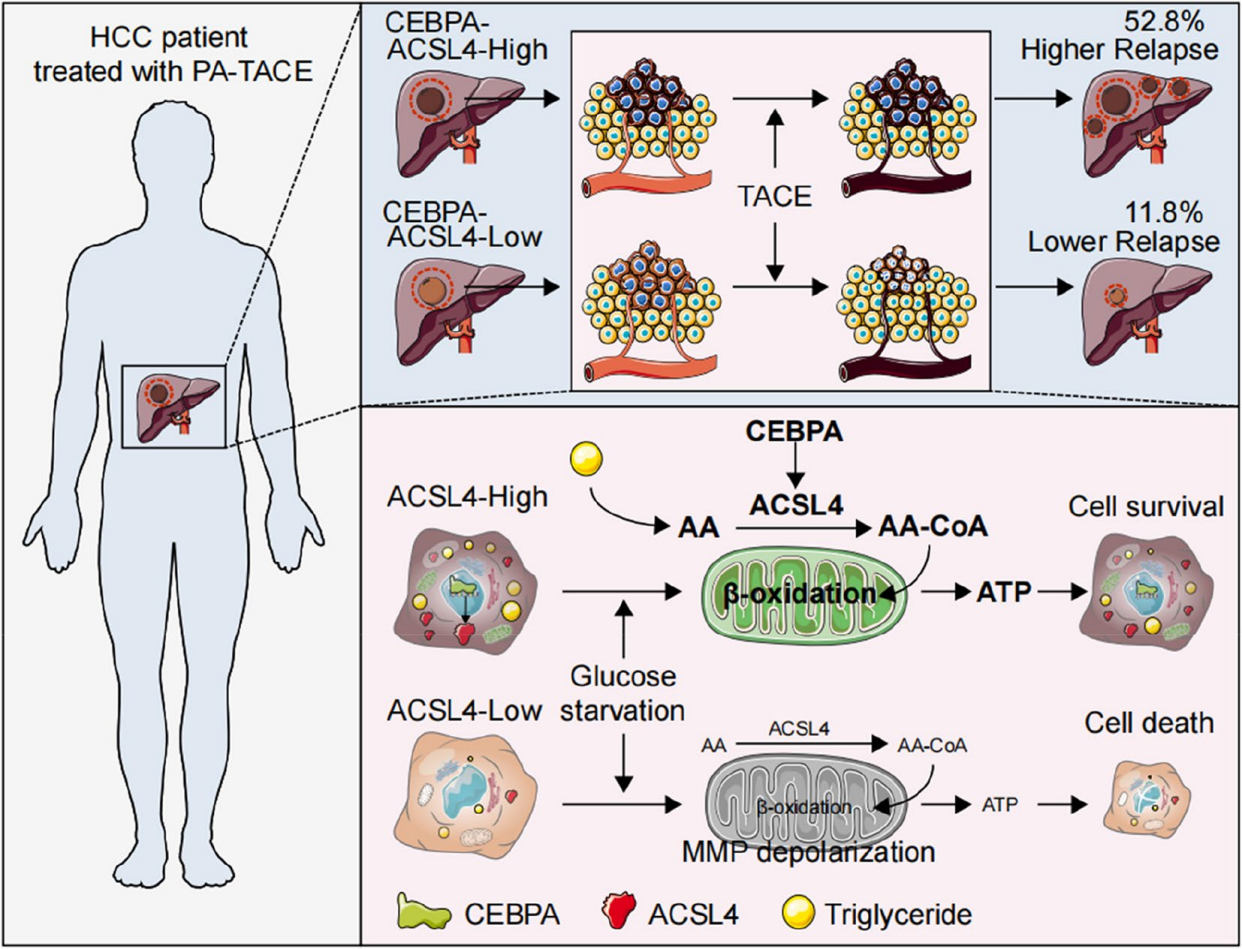

**Supplementary Information:**

The online version contains supplementary material available at 10.1186/s13046-024-03222-5.

## Highlights

1. Elevated CEBPA-ACSL4 levels in tumors links to adverse prognosis in HCC patients treated with PA-TACE.

2. ACSL4 shields HCC cells from TACE-triggered glucose starvation via AA-involved β-oxidation.

3. CEBPA up-regulates ACSL4 transcriptionally and protects HCC cells from cell death.

**Supplementary Information:**

The online version contains supplementary material available at 10.1186/s13046-024-03222-5.

## Introduction

Liver cancer, specifically hepatocellular carcinoma (HCC), poses a significant global health challenge, with a notable rise in incidence in previously low-risk Western countries. This trend correlates with the rise of metabolic diseases, including non-alcoholic fatty liver disease (NAFLD) and diabetes [1], which are often associated with abnormalities in lipid metabolism. There is growing evidence suggesting that lipid metabolism plays a critical role in HCC pathogenesis, key enzymes in certain lipid metabolic pathways, such as CCAAT/enhancer binding protein α (CEBPA) [2] and carnitine palmitoyltransferase 1 (CPT1) [3], are implicated in HCC by modulating lipid synthesis and oxidation, processes essential for tumor growth and survival.

Acyl-CoA synthetase long chain 4 (ACSL4), one of the ACSL family member that is crucial for fatty acid activation[4], is dysregulated in HCC and associated with poor outcomes of HCC patients [4, 5]. Our previous study reported that ACSL4-high expression in tumor tissue was correlated with good prognosis in HCC patients treated with sorafenib, due to ACSL4's role in promoting ferroptosis induced by sorafenib [6]. Despite its effect in lipid anabolic metabolism and pro-ferroptosis [6], the role of ACSL4 in promoting HCC and influencing patient survival in diverse contexts has not been fully elucidated yet.

Transarterial chemoembolization (TACE) and transarterial embolization (TAE) are commonly used to manage intermediate-stage HCC patients, with almost half of all HCC patients anticipated to undergo TACE/TAE treatment during the course of their disease [7, 8]. Clinically, TACE is also used as a postoperative adjuvant therapy to prevent the recurrence of HCC after surgical resection, which’s efficacy is supported by randomized controlled trials [9–11], though not all patients benefitted from this strategy [11].

Mechanistically, TACE exerts its therapeutic effects through ischemic embolization-induced hypoxia and the cytotoxicity of chemotherapeutic drugs. However, ischemia-induced hypoxia can up-regulate hypoxia-inducible factors (HIFs) [12], known drivers of cancer progression [13]. Discrepancies between TACE and TAE outcomes suggest a potential redundancy of chemotherapeutic agents in TACE [14, 15], highlighting the need for a deeper understanding of the molecular mechanisms governing TACE efficacy.

This study aims to elucidate the clinical relevance of ACSL4 in HCC patients undergoing PA-TACE and uncover how ACSL4 protects cell survival against cell death caused by glucose deprivation. By shedding light on these mechanisms, we strive to enhance our understanding of HCC progression and treatment responses, paving the way for improved therapeutic strategies in the management of this challenging disease.

## Materials and methods

A full list of materials and detailed methods are available in the Supplementary Materials & Methods.

### HCC cohorts

The studies involving two HCC cohorts. In the first Singapore cohort, a total of 202 primary HCC patients who underwent curative resection at the National University Hospital of Singapore were included. Tissue samples from these patients were collected and arranged into a tissue microarray. The expression levels of ACSL4 protein were assessed through immunohistochemistry (IHC) staining using a diluted mouse monoclonal antibody from Santa Cruz Biotechnology. The scoring was based on a four-tiered grading system, while CEBPA expression was evaluated according to a previous study [2]. A final cohort of 187 HCC patients was considered for survival analysis.

The second China-Guangxi cohort consisted of two groups of HCC patients. The first group comprised 70 primary HCC patients who underwent resection as the primary treatment and TACE as postoperative adjuvant therapy at the First Affiliated Hospital of Guangxi Medical University. The second group included 10 recurrent HCC patients who experienced recurrence within 2 years after curative resection and subsequently received TACE as the primary post-recurrence treatment. The expression levels of ACSL4 and CEBPA proteins were quantified based on the average optical density (AOD). The cohort was divided into ACSL4-high and ACSL4-low groups based on the median AOD value of ACSL4. Similarly, the CEBPA-positive rate was calculated, and patients were categorized into CEBPA-high and CEBPA-low groups using a predefined criterion. The clinicopathological characteristics of both cohorts are detailed in Table S1-S3.

### Cell culture and treatment

Human Huh7, Hep3B, HCC-M, HepG2, PLC/PRF/5 (PLC/5), SNU-398, and rat N1S1 cell lines were obtained from authenticated cell culture repositories. The cells were cultured in specific media supplemented with 10% FBS (Gibco, Newcastle, Australia) and subjected to nutrient starvation as required. Cell viability was assessed using a propidium iodide exclusion assay in a flow cytometer (CytoFLEX, Beckman, Atlanta, USA), as previously [2].

### Metabolic assays and recovery study with fatty acids supplementation

Biochemical or fluorescent metabolic assays, such as ATP, Oil Red O, Nile red, triglyceride, and *β*-hydroxybutyrate were performed according to the provided protocols by the respective manufacturers. Recovery studies were performed in the glucose starved HCC cells by supplementation with Bovine serum albumin (BSA)-conjugated oleic acid (OA), Arachidonic acid (AA) or other types of fatty acids or Arachidonoyl coenzyme A (AA-CoA).

### Ectopic HCC xenograft experiments

Male BALB/c nude mice from Hunan SJA Laboratory Animal (Changsha, China) were used for the xenograft experiments. For ACSL4-knockout and ACSL4-overexpressing experiments, 32 and 36 mice were inoculated with specific cell lines (1 × 10^6^ cells) subcutaneously into the right flank and treated with PBS or canagliflozin (30 mg/kg, once every other day) by gavage. Tumor growth was monitored, and relevant measurements were taken post-sacrifice. Immunohistochemistry experiments were performed on collected tumor samples [2].

### Statistical analyses

Statistical analyses were carried out using SPSS software version 22.0. Various tests such as one-way ANOVA, Kruskal–Wallis test, *t*-tests, *Chi*-Square tests, and correlation analyses were used to analyze experimental data. Survival analyses of HCC patients were conducted using Kaplan–Meier and multivariate Cox regression models. Data are presented as mean ± SD from at least three independent experiments, with statistical significance set at *P* < 0.05.

## Results

### ACSL4 expression is upregulated in HCC and correlates with poor patient survival

**Fig. 1:**
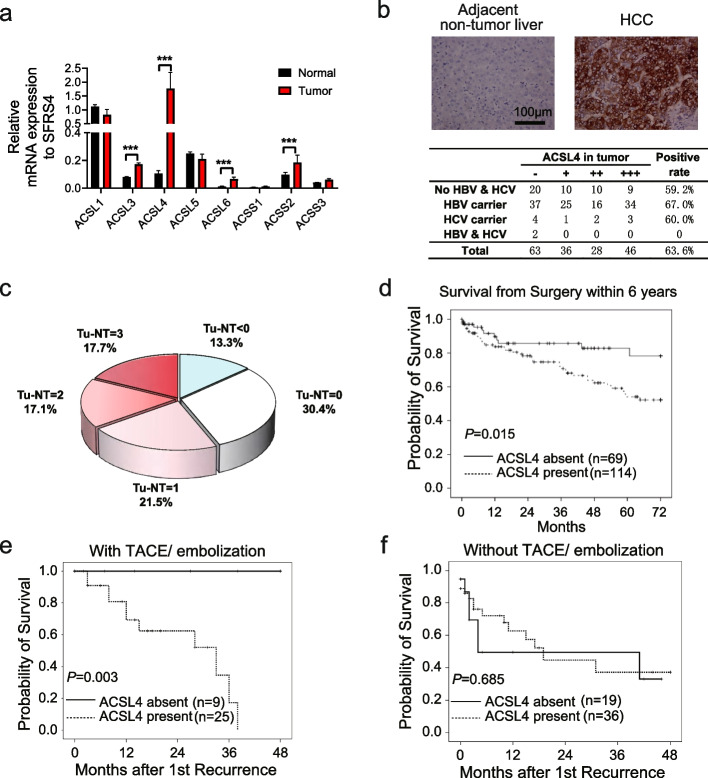
ACSL4 up-regulation in HCC was associated with poorer survival particularly among those recurrent patients who had TACE as post-recurrent treatment. **a** Expression of ACSL and ACSS family members were determined by qRT-PCR in HCC and adjacent normal liver tissues (*N* = 20). **b** ACSL4 was determined by IHC in tissue microarrays collected from the Singapore HCC cohort (*N* = 170). The expression levels were divided according to a four-tiered grading system. **c** A pie chart of the difference of ACSL4 protein expression between non-tumor (NT) and HCC tumor tissue (Tu). **d** A Kaplan–Meier survival comparison between ACSL4-present and ACS4-abssent HCC patients. **e**–**f** Kaplan–Meier survival comparisons between ACSL4-present and ACSL4-absent recurrent HCC patients who had TACE/TAE as post-recurrent treatment (**e**) or who had other treatments except TACE/TAE (**f**). ***, *P* < 0.001

Among ACSL family members, ACSL4 was found to be significantly upregulated in HCC tissues compared to adjacent non-tumor tissues, as revealed by bioinformatic analyses (Fig. S1a-b) and transcriptional analysis (Fig. 1a). Further analyses of a HCC tissue microarray [2] from a Singapore HCC cohort confirmed that ACSL4 expression was present in approximately 60% of primary HCC tissues (Fig. 1b-c) and was associated with poorer overall survival in HCC patients (*P* = 0.015, Fig. 1d and *P* = 0.043, Fig. S2a). Higher expression of ACSL4 in HCC was correlated with elevated levels of serum alpha-fetoprotein (AFP) (*P* = 0.0004, Fig. S2b). ACSL4 also predicted poor patient survival among those AFP-low patients (< 200 ng/mL, Fig. S2c-d). Multivariate Cox analyses revealed that ACSL4 expression in HCC (hazard ratio [HR] = 4.229, 95% confidence interval [95%CI] = 1.189 ~ 15.042), TNM staging (HR = 5.704, 95%CI = 1.719 ~ 18.923), and early recurrence within 2 years (HR = 2.394, 95%CI = 1.032 ~ 5.555) are independent predictors of overall survival in HCC patients (Table 1). Different treatment options were applicable for these recurrent patients, dependent on stages of recurrent HCC and functions of remaining liver (Fig. S2g). Notably, ACSL4 expression inversely correlated with the presence of tumor necrosis in HCC tissues (*P* = 0.007, Table S1-S2), suggesting a potential role of ACSL4 in tumor physiology and response to therapy.

**Table 1:** Tumor ACSL4 expressions are independently associated with poor HCC overall survival

Variables	Number of cases	Univariate Cox analysis		Multivariate Cox analysis	
		**Crude HR (95% CI)**	***P*-value**	**Adjusted HR (95% CI)**	***P*-value**
**ACSL4 in tumor** (absent *vs.* present)	69 / 114	2.306 (1.153 ~ 4.611)	**0.018**	4.229 (1.189 ~ 15.042)	**0.026**
Age (< 50 *vs.* ≥ 50)	47 / 135	0.969 (0.515 ~ 1.824)	0.922		
Gender (male *vs.* female)	150 / 37	1.876 (1.024 ~ 3.436)	**0.042**	1.217 (0.474 ~ 3.122)	0.683
HBV (no *vs.* yes)	60 / 114	0.931 (0.512 ~ 1.692)	0.814		
HCV (no *vs.* yes)	167 / 12	0.719 (0.175 ~ 2.961)	0.646		
BCLC (0&A *vs*.B)	91 / 28	1.383 (0.580 ~ 3.298)	0.465		
**Child–Pugh (A&B *vs*. C)**	101 / 16	3.124 (1.239 ~ 7.875)	**0.016**	2.568 (0.984 ~ 6.702)	**0.054**
Diabetes (no *vs.* yes)	133 / 47	0.528 (0.247 ~ 1.128)	**0.099**	1.465 (0.430 ~ 4.996)	0.542
Fatty Liver (no *vs.* yes)	163 / 24	0.860 (0.366 ~ 2.020)	0.729		
Fibrosis (no *vs.* yes)	151 / 36	0.588 (0.275 ~ 1.254)	0.165		
Cirrhosis (no *vs.* yes)	87 / 100	1.290 (0.737 ~ 2.258)	0.371		
Tumor size (< 5 *vs.* ≥ 5 cm)	89 / 95	1.564 (0.883 ~ 2.771)	0.122		
Number of nodules (single *vs.* multiple)	129 / 58	2.028 (1.147 ~ 3.584)	**0.015**	0.834 (0.340 ~ 2.046)	0.691
**TNM staging (I *vs.* II, III and IV)**	87 / 91	3.468 (1.885 ~ 6.382)	** < 0.001**	5.704 (1.719 ~ 18.923)	**0.004**
Tumor necrosis (no *vs*. yes)	93 / 93	1.038 (0.593 ~ 1.818)	0.895		
**Recurrence within 2 years** (no *vs*. yes)	115 / 72	5.889 (3.119 ~ 11.119)	** < 0.001**	2.394 (1.032 ~ 5.555)	**0.042**

### ACSL4 expression predicts post-recurrent survival in HCC patients undergoing TACE/TAE

ACSL4 expression did not significantly affect recurrence rates in HCC patients (*P* = 0.549, Fig. S2e); however, its presence in primary tumors was associated with poorer survival after recurrence, especially among those experiencing early recurrence within 2 years (*P* = 0.033, Fig. S2f). Notably, ACSL4 expression was significantly associated with worse survival outcomes in recurrent patients who underwent TACE/TAE as post-recurrent treatment (*P* = 0.003, Fig. 1e), but not in those receiving non-TACE/TAE treatments (Fig. 1f). The differential impact of ACSL4 presence was evident, with all ACSL4-absent patients (9/9) surviving 4 years post-TACE/TAE treatment compared to ACSL4-present patients who experienced gradual mortality within the same timeframe.

### ACSL4 is essential for HCC cell survival in response to glucose starvation in vitro

**Fig. 2:**
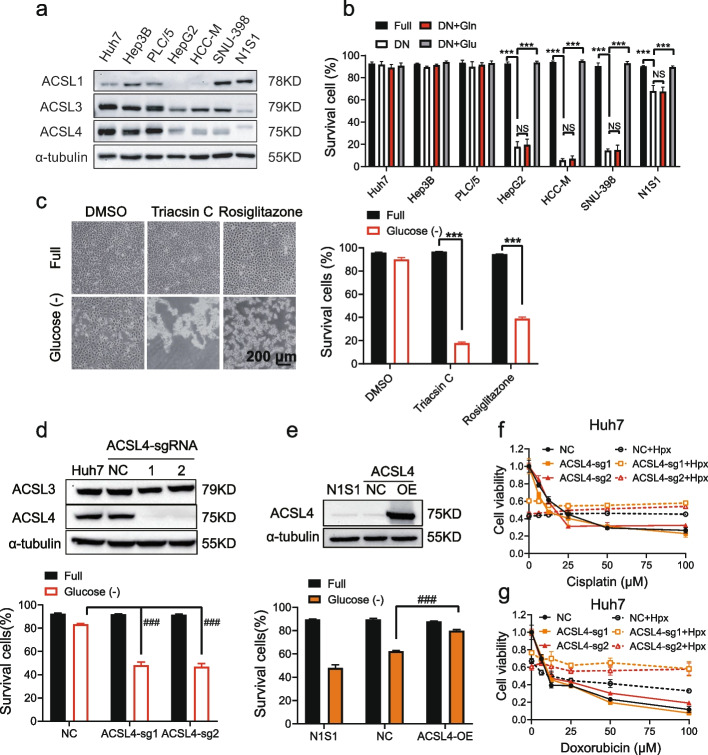
ACSL4 protected HCC cells against glucose starvation induced cell death. **a** ACSL1, ACSL3 and ACSL4 expression in 7 HCC cell lines. **b** The indicated HCC cells were treated with full medium (Full), glucose & glutamine double deprivation (DN), DN with 4 mM glutamine (Gln) or DN with 4.5 g/L glucose (Glu) for 24 h. The proportion of survival cells were determined by PI assay. **c** Huh7 cells were treated with ACSL4 inhibitor triacsin C (2 μM) or rosiglitazone (20 μM) in full medium or glucose starvation for 24 h. The proportion of survival cells is shown in the right panel. **d** ACSL4-knockout Huh7 cells (ACSL4-sg1, -sg2, upper panel) were treated with full medium or glucose starvation for 60 h. The proportion of survival cells is shown in the lower panel. **e** ACSL4-overexpressing N1S1 cells were treated with full medium or glucose starvation for 36 h. **f-g** ACSL4-knockout huh7 cells were treated with cisplatin (**f**) or doxorubicin (**g**) with or without hypoxia (Hpx) for 36 h or 24 h respectively. ###, compared with negative control (NC), *P* < 0.001; **, *P* < 0.01; ***, *P* < 0.001; NS, not significant

TACE treatment causes ischemic and cytotoxic effects, both of which can induce HCC cell death. Ischemia involves nutrient deprivation and hypoxia. Following our observations on TACE treatment effects, we investigated whether ACSL4 modulates cell survival under the resultant cellular stresses, specifically ischemia and cytotoxic effects. ACSL4-low-expressing (ACSL4-low) human HepG2, HCC-M, SNU-398 cell lines and rat N1S1 cell lines were sensitive to glucose- and glutamine-double starvation, while ACSL4-high-expressing (ACSL4-high) Huh7, Hep3B, and PLC/5 cells were not as affected (Fig. 2a-b). Glucose supplementation rescued induced cell death (Fig. 2b), highlighting glucose starvation as the key pro-death stress.

The essential role of ACSL4 was confirmed through a series of experiments. Inhibition of ACSL4 enzyme activity sensitized cells to glucose starvation-induced death (Fig. 2c), while ACSL4 knock-down and knockout increased cell death sensitivity (Fig. S3a and 2d). Conversely, ACSL4 overexpression rescued ACSL4-low cells from glucose starvation-induced death (Fig. 2e and S3b).

In contrast to its protective effects against glucose starvation, ACSL4 did not impact cell death in response to hypoxia alone or in combination with cisplatin or doxorubicin (two common chemotherapeutic drugs used in TACE) in glucose-replete conditions (Fig. 2f-g and S3c-e). Additionally, ACSL4 promoted clone formation, migration, and invasion of HCC cells (Fig. S3f-n). In addition, ACSL4 expression was stable independent of glucose starvation treatment (Fig. S3o). These findings collectively suggest that ACSL4 is crucial for HCC cell survival during glucose starvation.

### ACSL4 promotes fatty acid catabolism to support starved HCC cells

**Fig. 3:**
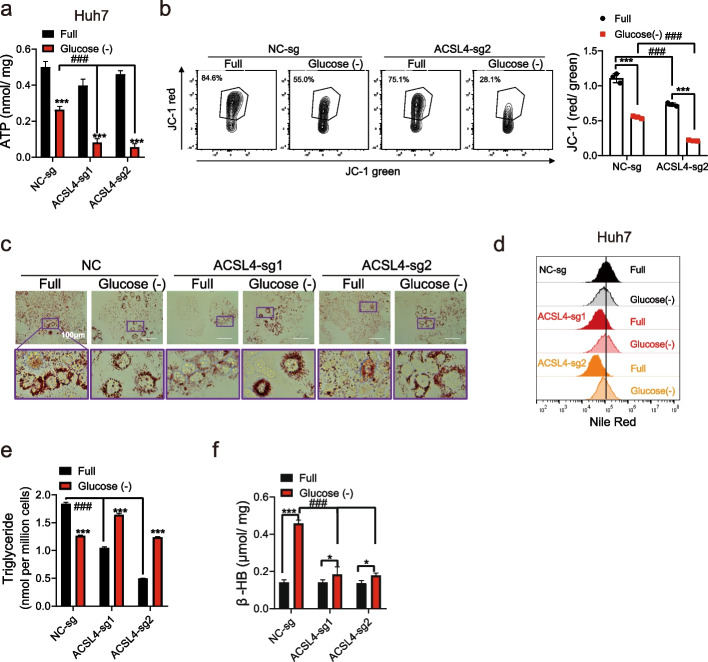
ACSL4-KO obstructed maintenance of cellular ATP levels by inhibiting *β*-oxidation under glucose starvation. **a** ACSL4-KO Huh7 cells were treated with glucose starvation for 48 h. Then cellular ATP were detected. **b** ACSL4-KO Huh7 cells were treated with glucose starvation for 12 h, mitochondrial membrane potential was detected using JC-1 dye by flow cytometry. The ratios of red/green florescence were shown. **c-e** ACSL4-KO Huh7 cells were treated with glucose starvation for 24 h. The cellular lipid droplets were stained with oil red O dye (**c**) or Nile red dye (**d**), Nile red fluorescence was assessed by flow cytometry. The cellular triglycerides were detected (**e**). **f** ACSL4-KO Huh7 cells were treated with glucose starvation for 48 h. Then cellular *β*-hydroxybutyrate (*β*-HB) were detected. ###, compared with NC, *P* < 0.001; ***, *P* < 0.001

Glucose starvation caused more ATP depletion in ACSL4-KO cells than in control cells (Fig. 3a), while cells overexpressing ACSL4 maintained stable ATP levels (Fig. S4a-b). It was reported that ACSL4 was required for the maintenance of mitochondrial membrane potential (MMP) in chemo-resistant triple-negative breast cancer (TNBC) cells [16]. Consistently, ACSL4 was found to be essential for maintaining MMP in HCC cells, especially under glucose starvation conditions (Fig. 3b and S4c). ACSL4 was involved in fatty acid activation, essential for both lipid anabolism and catabolism. Under nutrient-replete conditions, ACSL4-KO cells contained fewer lipid droplets and triglycerides than controls (Fig. 3c-e and S4d). Surprisingly, glucose starvation induced an unexpected accumulation of lipid droplets and triglycerides in ACSL4-KO cells, in contrast to control cells where these levels decreased. Consistently, ACSL4 overexpression exhibited opposite effects (Fig. S4e-f). These results suggest that the role of ACSL4 switches from lipid biosynthesis to lipid catabolism during glucose starvation.

### ACSL4 induces fatty acid activation and β-oxidation to promote cell survival

**Fig. 4:**
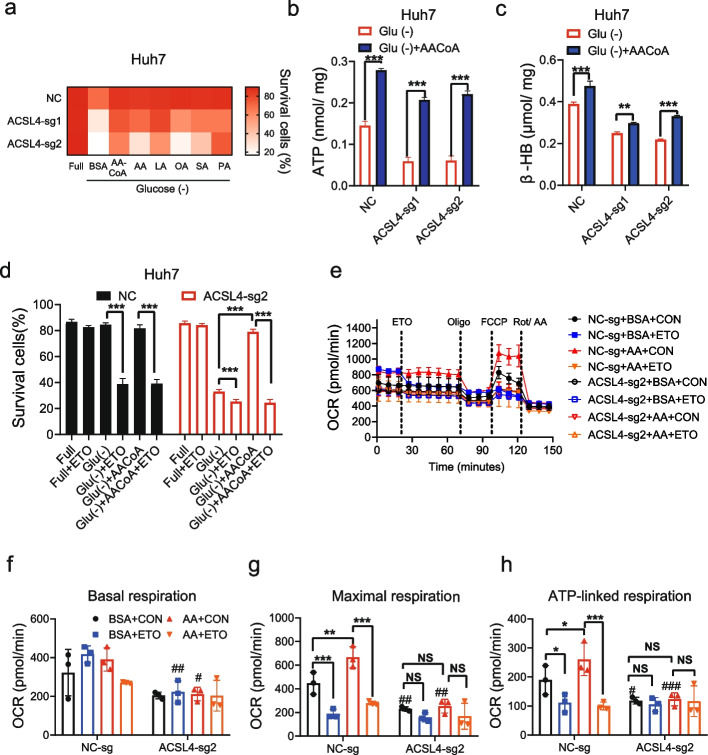
ACSL4 switched on the utilization of AA in *β*-oxidation to supply energy. **a-c** ACSL4-KO Huh7 cells and negative control cells were treated with glucose starvation medium with/without BSA or different BSA-fatty acid conjugates (AA-CoA, AA, LA, PA, OA, SA; 25 μM for each) for 60 h. Then the proportion of survival cells (**a**), cellular ATP (**b**) and *β*-HB (**c**) were detected. **d** ACSL4-KO Huh7 cells were treated with glucose starvation medium supplied with AA-CoA (25 μM) and/or etomoxir (ETO, 40 μM). The proportion of survival cells were detected. **e–h** ACSL4-KO Huh7 cells were treated with 25 μM AA or BSA after incubated in glucose-limited medium for 12 h. Then mitochondrial OCRs were determined by a Seahorse XF analyzer (**e**). The basal OCR (**f**), ATP-linked OCR (**g**) and maximal OCR (**h**) were summarized, respectively. Abbreviations: BSA, bovine serum albumin; AA-CoA, arachidonoyl coenzyme A; AA, arachidonic acid; LA, linoleic acid; OA, oleic acid; PA, palmitic acid; SA, stearic acid. ETO, etomoxir. # compared with NC, *P* < 0.05; ##, compared with NC, *P* < 0.01; ###, compared with NC, *P* < 0.001; **, *P* < 0.01; ***, *P* < 0.001; NS, not significant

Triglyceride catabolism releases fatty acids, which are further broken down via *β*-oxidation mainly in mitochondria and peroxisomes to generate acetyl-CoA for mitochondrial ATP production. Following glucose starvation, ACSL4-high cells demonstrated significantly increased levels of *β*-hydroxybutyrate (*β*-HB) (Fig. 3f and S4g), a direct indicator of enhanced fatty acid oxidation mediated by ACSL4. The rescue of ACSL4-low cells from glucose starvation-induced death by supplementation with activated AA-CoA, rather than its precursor, underscores ACSL4's specific role in the crucial activation step of fatty acid metabolism (Fig. 4a and S5a-b). Consistently, AA-CoA supplementation promoted the generation of ATP and *β*-HB (Fig. 4b-c and S5c-d). In contrast, inhibition of *β*-oxidation using etomoxir sensitized ACSL4-high cells to glucose starvation-induced cell death (Fig. 4d and S5e).

Furthermore, we tested the mitochondrial oxygen consumption rates (OCR) through fatty acid *β*-oxidation (Fig. 4e-h and S5f-g). After glucose starvation, the ACSL4-high HCC cells had higher ATP-linked OCR and higher maximal OCR. However, these effects were decreased or abolished by etomoxir pretreatment. Critically, while arachidonic acid (AA) supplementation significantly boosted both ATP-linked and maximal oxygen consumption rates (OCR) in ACSL4-high cells, these enhancements were not observed in ACSL4-low cells, illustrating the dependency of metabolic efficiency on ACSL4 expression levels (Fig. 4e-h and S5f-g). Again, etomoxir pretreatment abolished this effect. Taken together, these results highlight the importance of ACSL4-mediated fatty acid activation and subsequent *β*-oxidation for HCC cell survival during glucose starvation.

### Canagliflozin mimics glucose restriction and inhibits ACSL4-low tumors in vivo

**Fig. 5:**
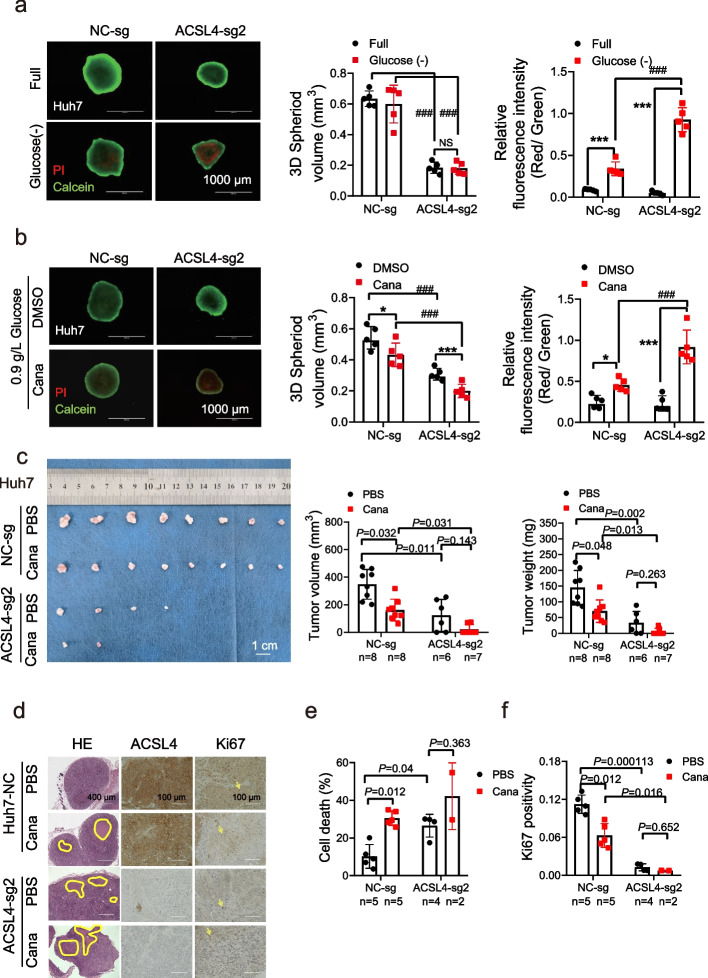
Canagliflozin inhibited ACSL4-KO HCC cells by mimicking glucose restriction in vitro and in vivo. **a-b** The 3D Spheroid tumor developed by indicated cells were treated with glucose starvation (**a**) or canagliflozin (Cana, 20 μM) in glucose-present (0.9 g/L) medium (**b**) for 60 h. The dead cells were stained with PI (red fluorescence) while live cells were stained with calcein (green fluorescence) before observation under a fluorescent microscopy (left panel). The volume of spheroid (middle panel) and cell death (the ratio of red to green, right panel) were measured.** c** The mice bearing xenograft developed from ACSL4-KO or control Huh7 cells were treated with control PBS or Cana (30 mg/kg) for 33 days once every other day. The resected tumors were photographed (left panel). The tumor volumes (middle panel) and tumor weights (right panel) were measured. **d-f** Representative images of hematoxylin & eosin staining (HE), IHC for ACSL4 and Ki67 are shown (**d**). The proportion of area of necrotic tissue (**e**) and the Ki-67 positive score (**f**) were summarized. ##, compared with NC, *P* < 0.01; ###, compared with NC, *P* < 0.001; ***, *P* < 0.001. NS, not significant

Consistent with observations in 2D cell culture systems, ACSL4-low cell-derived 3D spheroids exhibited a similar increased susceptibility to glucose starvation-induced cell death, reinforcing the model's validity (Fig. 5a and S6a). To simulate glucose starvation in a clinically relevant manner, we employed canagliflozin, an anti-diabetes drug known for inhibiting sodium-glucose transport protein 2 (SGLT2), effectively reducing glucose intake by cells [17]. Treatment with canagliflozin in glucose-low (0.9 g/L) medium resulted in significantly higher levels of spheroid cell death in ACSL4-low cells (Fig. 5b, S6b) or ACSL4-overexpression cells treated with ACSL4 inhibitor (Fig. S6c).

We then performed subcutaneous tumor inoculation to replicate reduced blood supply conditions, closely mimicking the ischemic environment induced by TACE, and assessed the drug’s efficacy in this context. The efficacy of canagliflozin in inhibiting ACSL4-high or-low HCC xenograft tumors was evaluated in vivo. Systemic administration of canagliflozin led to a moderate decrease in serum glucose levels to approximately 17% below baseline after 18 h of treatment (Fig. S6d-e). Notably, canagliflozin treatment resulted in a substantial reduction (60–80%) in glycogen content within the tumor nodules (Fig. S6f), without affecting the body weight of the mice (Fig. S6g-h).

Canagliflozin treatment more effectively suppressed tumor growth in mice inoculated with ACSL4-low cells compared to those with ACSL4-high cells (Fig. 5c-f and S6i-l). Notably, ACSL4-KO inhibited the growth of tumor and canagliflozin further hindered the growth of ACSL4-KO xenografts (Fig. 5c). In addition, ACSL4-KO increased necrosis in tumors (Fig. 5d-e) and canagliflozin induced larger areas of necrosis in the nodular core (Fig. 5d-e and S6j-k) and inhibited cell proliferation (Fig. 5d, f and S6k, l), particularly in ACSL4-low xenografts. In contrast, ACSL4 overexpression reversed these effects. These results collectively demonstrate that canagliflozin partially mimics glucose restriction in the tumor microenvironment, making ACSL4-low tumors more susceptible to regression than ACSL4-high tumors.

### CEBPA transcriptionally activates ACSL4 and protects HCC cells

**Fig. 6:**
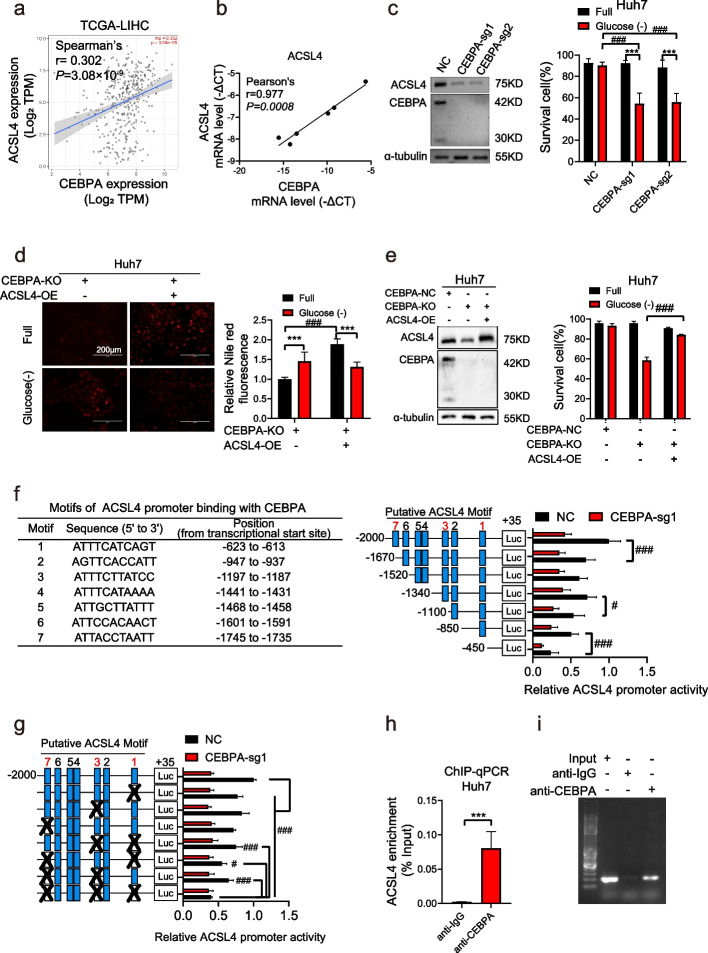
ACSL4 is positively regulated by transcriptional factor CEBPA. **a** Correlation of mRNA expression between CEBPA and ACSL4 in HCC (Timer 2.0 online database, http://timer.cistrome.org/). **b** Correlation of mRNA expression between CEBPA and ACSL4 in 6 HCC cells. **c** CEBPA-knockout Huh7 cells (CEBPA-sg1, CEBPA-sg2) and its negative control (NC) were treated with glucose starvation for 24 h. The proportion of survival cells is shown in the right panel. **d** Indicated cells were treated with glucose starvation for 12 h, the cellular lipid droplets were stained with Nile red dye. **e** CEBPA-sg1 cells (CEBPA-KO) were transfected with ACSL4-overexpression (ACSL4-OE) lentivirus, then were treated with glucose starvation for 24 h. The proportion of survival cells is shown in the right panel. **f** Putative motifs of ACSL4 promoter binding with CEBPA predicted by JASPAR database (https://jaspar.elixir.no/). Indicated cells were transfected with a variety of truncated ACSL4 promoter constructs, and the luciferase activity normalized by Renilla luciferase activity were determined. **g** Indicated cells were transfected with single or simultaneous deletion mutated ACSL4 promoter plasmids at motif 1, 3, 7, the relative luciferase activity was determined. **h-i** ChIP-qPCR assays to measure the enrichment of ACSL4 promoter on CEBPA or IgG in Huh7 cells (**h**). PCR products were detected by agarose gel electrophoresis (**i**)

Our previous investigations revealed that the upregulation of CEBPA, a transcription factor involved in glucose and lipid metabolism, was associated with poor survival in HCC patients and conferred protection against glucose starvation [2]. Interestingly, CEBPA expression exhibited a specific correlation with ACSL4, rather than other members of the ACSL family, in TCGA-LIHC samples and various HCC cell lines (Fig. 6a-b and Fig. S7). Knockout or enforced overexpression of CEBPA further confirmed its specific regulatory role in ACSL4 expression (Fig. 6c and S8a). Moreover, knockout of CEBPA rendered cells susceptible to glucose starvation-induced cell death (Fig. 6c), while CEBPA overexpression exerted the opposite effect (Fig. S8a). The enforced expression of ACSL4 in CEBPA-knockout cells rescued lipid catabolism (Fig. 6d) and prevented induced cell death (Fig. 6e), underscoring the crucial role of ACSL4 in cell survival under glucose starvation.

Luciferase reporter assays demonstrated consistent ACSL4 promoter activity in CEBPA-high cells, but not in CEBPA-low cells (Fig. S8b-c). Bioinformatic analyses using JASPAR databases identified seven putative binding motifs of CEBPA in the upstream region of the ACSL4 locus (Fig. 6f). Specifically, reporter constructs containing motif 1 (-623 to -613), motif 3 (-1197 to -1187), and motif 7 (-1745 to -1735) were responsive to CEBPA-mediated transcription (Fig. 6f). Deletions of these three responsive motifs or their combination further confirmed their involvement in CEBPA-mediated ACSL4 transcription (Fig. 6g). Additionally, the physical binding between the CEBPA protein and the promoter region of ACSL4 was validated using ChIP-qPCR assays (Fig. 6h-i). These findings collectively demonstrate that CEBPA promotes cell survival through its transcriptional regulation of ACSL4.

### ACSL4 and CEBPA predicate poor treatment responses among HCC patients undertaking PA-TACE

**Fig. 7:**
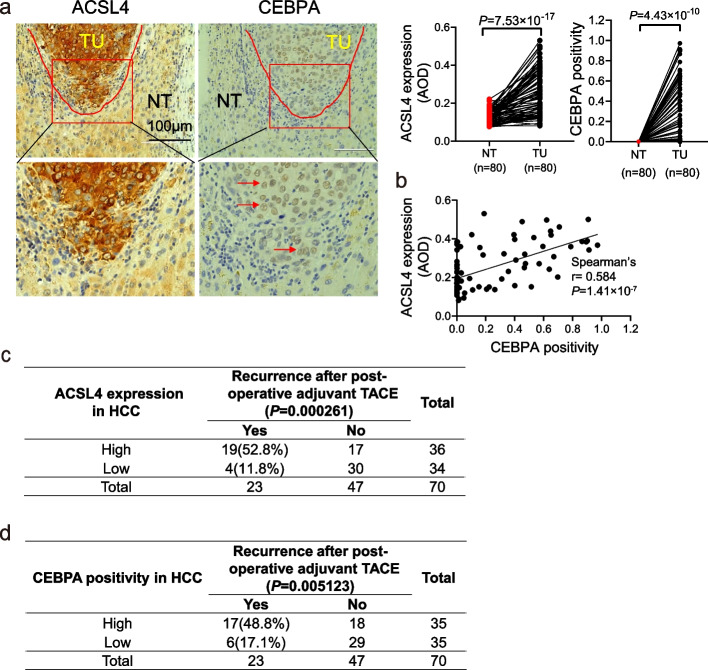
Higher ACSL4 and CEBPA expression in HCC was associated with higher recurrence of HCC patients with TACE treatment. **a** ACSL4 protein expression (measured as the average optical density (AOD) using ImageJ software) and CEBPA positivity (the ratio of CEBPA-positive cells to all cells) in HCC (TU) and adjacent nontumor (NT) tissue samples of Guangxi cohort (*n* = 80) were summarized on the middle and right panel respectively. The representative pictures were shown in the left panel, CEBPA-positive cells were marked using red arrow. **b** The correlation between ACSL4 and CEBPA in expression of HCC tissue samples in Guangxi cohort. **c-d** Comparison of recurrence between ACSL4-High and ACSL4-Low HCC patients (**c**) or CEBPA-High and CEBPA -Low HCC patients (**d**) who received PA-TACE treatment

To assess the potential utility of ACSL4 and CEBPA as prognostic markers for TACE treatment, we analyzed a second cohort of HCC patients from Guangxi. Consistent with the results from the Singapore HCC cohort, HCC patients in the Guangxi cohort exhibited higher expression of ACSL4 in HCC tissues compared to adjacent non-cancerous tissues (*P* < 0.001, Fig. 7a). Additionally, CEBPA was up-regulated in HCC tissues (*P* < 0.001, Fig. 7a and S8d). Importantly, CEBPA protein expression positively correlated with ACSL4 expression (Fig. 7b).

Following PA-TACE treatment, 52.8% (19/36) of patients with ACSL4-high expression experienced relapse within one year, in contrast to 11.8% (4/34) of those with ACSL4-low expression (*P* < 0.001, Fig. 7c). A similar trend was observed for CEBPA-high patients, with 48.8% experiencing relapse compared to 17.1% of CEBPA-low patients (*P* = 0.005, Fig. 7d). In a subset of recurrent patients treated with TACE post-recurrence, all patients with high ACSL4 expression (4/4) experienced another recurrence within a year, contrasting with only 33.3% of those with low expression (2/6). Although not statistically significant (*P* = 0.076, Fig. S8e), this trend suggests a potential pattern that warrants further investigation, possibly limited by the small sample size. A similar trend was also observed between CEBPA-high and CEBPA-low patients (Fig. S8f). These results collectively indicate that ACSL4 and CEBPA expression in HCC tissues could serve as valuable prognostic indicators for the response to TACE treatment.

## Discussion

The rising incidence of HCC in formerly low-risk Western countries is attributed to the escalating prevalence of metabolic diseases like obesity and diabetes [1]. Obesity, a known risk factor for various cancers, is linked to increased cancer mortality, with liver cancer showing the highest relative risk among men [18]. The critical role of lipid metabolism in liver cancer is further underscored by the association of NAFLD with HCC development. Studies have highlighted the upregulation of hepatic ACSL4 in NAFLD patients, with ACSL4 suppression yielding beneficial effects on liver steatosis and fibrosis in preclinical models [19].

Prior research has identified ACSL4 as an oncogene in various cancers, including HCC [5, 20–22]. In our experiments, ACSL4 overexpression promoted tumor proliferation in 2D cell culture models (Fig. S3j), yet this effect was not observed in xenograft animal models (Fig. S6i). In 2D culture, cells are exposed to a uniform supply of nutrients and oxygen, promoting cell proliferation. ACSL4 overexpression may enhance this growth by facilitating lipid biosynthesis, consistent with previous reports [5]. In contrast, when transplanted subcutaneously, tumor cells face limitations in nutrient and oxygen availability, which may hinder their proliferation [23, 24]. While ACSL4 overexpression may confer some advantages, it may not fully compensate for these environmental constraints, leading to the observed lack of significant tumor size differences in the PBS-treated ACSL4 overexpression group compared to controls.

The prognostic value of ACSL4 remains complex and somewhat controversial, as studies report conflicting outcomes regarding its impact on patient survival and treatment response. While some studies link ACSL4 upregulation to poorer patient outcomes through c-Myc pathway [5, 25], others suggest a potential benefit of its pro-ferroptosis manner in response to specific treatments like sorafenib [6]. In our study involving two distinct patient cohorts, heightened ACSL4 expression correlates with poorer overall survival, particularly among HCC patients undergoing PA-TACE treatments (Fig. 1 and 7). This dichotomy underscores the complexity of ACSL4's role in HCC and hints at its potential utility as a predictive biomarker for personalized treatment selection. Patients with varying levels of ACSL4 expression in their primary HCC tissues may benefit from tailored treatment approaches, highlighting the need for further validation through randomized clinical trials to translate these findings into clinical practice effectively.

The ongoing quest to identify prognostic biomarkers and develop predictive algorithms for enhancing the efficacy of TACE/TAE in individual HCC patients reflects the growing emphasis on personalized treatment strategies. Currently, prognostic biomarkers in HCC tissues and serum, along with predictive algorithms based on clinicopathological parameters, are being explored to refine treatment approaches [26–29]. These efforts have classified prognostic biomarkers into two major categories based on their underlying molecular mechanisms.

The first category includes biomarkers related to angiogenesis and metastasis pathways, such as miR-125b [30], CD24, erythropoietin (EPO), OTUD7B [31], IL-6/STAT3 [32], and hypoxia inducible factor 1 alpha (HIF-1*α*)[33], associated with post-TACE recurrence and poorer prognosis after PA-TACE. These biomarkers modulate angiogenesis and tumor metastasis, highlighting their potential as indicators of treatment response and outcomes. The second category includes biomarkers associated with metabolic pathways, such as pyruvate kinase M2 (PKM2), whose inhibition shows promise in reversing therapeutic resistance to TACE [34]. This emphasizes the significance of metabolic pathways in dictating treatment response and patient prognosis.

This study elucidates the CEBPA-ACSL4-lipid catabolism pathway in TACE treatment, highlighting the mechanistic role of CEBPA-ACSL4 in inducing lipid catabolism and protecting HCC cells from glucose starvation-induced cell death. To address the challenge of managing ACSL4-high HCC patients undergoing TACE, strategies to inhibit ACSL4 activity, such as utilizing rosiglitazone, a clinically available drug for type II diabetes mellitus, are proposed. Additionally, a novel approach called Transarterial Sorafenib-Embolization (TASE), which combines TAE with sorafenib to synergistically induce cell death, presents a promising avenue for enhancing treatment efficacy [35, 36]. Mechanistically, this approach leverages glucose starvation effects on glycolysis and the inhibitory action of sorafenib on electron transport chain complex II and III, leading to the cessation of energy production and mitophagy.

ACSL4 plays a critical role in both glucose-deprived and glucose-sufficient conditions, although its specific functions differ based on metabolic context. In glucose-sufficient environments, ACSL4 promotes malignant progression of HCC via stabilization of c-Myc/SREBP1 or enhancement of PAK2 transcription [5, 22], sustaining cell growth via de novo lipogenesis. Conversely, under glucose-deprived conditions, our study highlights that ACSL4 becomes essential for survival by enabling a metabolic shift toward fatty acid oxidation and facilitating mitochondrial β-oxidation, thereby preventing cell death. Our clinical data further support this notion, as HCC tissues lacking ACSL4 exhibit increased susceptibility to necrosis under glucose deprivation (Table S1). Fatty acid activation is integral to both lipid anabolism and catabolism, and from this perspective, ACSL4's role remains consistent—it catalyzes the conversion of long-chain polyunsaturated fatty acids (such as arachidonic acid, AA) into CoA derivatives (acyl-CoA). These CoA derivatives act as key precursors that participate in either lipid synthesis or lipid degradation, with their specific metabolic pathway determined by external nutrient availability [4]. In summary, while ACSL4 consistently facilitates fatty acid activation, its impact on metabolic state transitions is profoundly influenced by the external environment and its interactions with various metabolic pathways and regulatory proteins.

Our mechanistic studies identify ACSL4 as a critical factor for HCC cell survival under energy impairment conditions, emphasizing its essential role in lipid metabolism and cell survival. The transcriptional activation of ACSL4 by CEBPA and its association with the pro-survival effects of CEBPA further underscore the significance of the CEBPA-ACSL4-lipid catabolism pathway in HCC cell survival. Previous research has demonstrated that starved MEF cells also accumulate lipid droplets in response to combined deficiency of amino acids, glucose and serum in Hank’s balance salt solution [37]. The increased lipid contents are likely derived from the release of fatty acids through autophagic digestion of cellular membrane lipids. Subsequent investigations have revealed that endoplasmic reticulum (ER)-residing diacylglycerol acyltransferase 1 (DGAT1) facilitates the mobilization of lipids from autophagy to newly-formed, clustered lipid droplets, and functions to mitigate lipotoxic deregulation of mitochondria [38]. To validate the essential role of autophagy, we treated starved cells with the lysosome inhibitor chloroquine (CQ). The results consistently demonstrated that CQ treatment abolished glucose starvation-induced lipid droplet biogenesis (Fig. S4d). Therefore, these findings highlight the essential role of ACSL4 in lipid metabolism and cell survival.

There are limitations to this study. Firstly, as tissue biopsies are infrequently performed in patients opting for TACE as first-line therapy, the practical applicability of our findings is more relevant to patients treated with PA-TACE than to all TACE-treated patients. In clinical practice, plasma miRNAs can serve as diagnostic indicators for liver cancer [39]. Therefore, for patients without available tissue biopsies, future studies could investigate the expression consistency of miRNAs targeting ACSL4, such as miR-211-5p [40], in both HCC tissue and plasma. This approach would enable the reflection of ACSL4 expression levels in liver cancer tissues by detecting miRNAs in plasma. Secondly, our approach combines the subcutaneous tumor model with canagliflozin treatment to simulate post-TACE conditions. Subcutaneous tumors, which develop in tissues with less developed blood vessel networks, experience lower blood flow compared to tumors in the highly vascularized liver environment [41]. Importantly, we observed that blood glucose levels in mice were reduced by canagliflozin treatment (Fig. S6d-e), further decreasing the blood glucose supply to subcutaneous tumors. While the model does not recreate all the complex metabolic processes of the liver, it is specifically designed to simulate the critical conditions of nutrient deprivation and hypoxia associated with reduced blood supply post-TACE. Thus, this subcutaneous model remains relevant for investigating the effects of glucose deprivation on tumor biology, particularly under conditions of ischemia following embolization. Thirdly, while our study has shed light on the mechanistic underpinnings of the CEBPA-ACSL4-lipid catabolism pathway, further research is warranted to fully understand the precise mechanisms governing the upregulation of ACSL4. Moreover, future studies involving TACE application in animal models with primary HCC [42] may contribute to expediting the clinical implementation of targeting the involved CEBPA-ACSL4-lipid catabolism pathway in HCC treatment.

## Conclusion

In summary, the upregulation of CEBPA and ACSL4 in HCC tissues is correlated with poorer patient survival and predicts a dismal response to PA-TACE treatment. Mechanistically, ACSL4 plays a dual role in HCC cells by promoting lipid accumulation under nutrient-rich conditions and enhancing lipid catabolism and energy production to support cell survival during glucose deprivation. The transcriptional upregulation of ACSL4 by CEBPA mediates the pro-survival effects of CEBPA. Therefore, ACSL4 and its upstream regulator CEBPA serve as promising prognostic indicators and potential therapeutic targets for TACE treatment in HCC, emphasizing the need for further research to fully understand the implications of this pathway for HCC management.

## Supplementary Information

Supplementary Material 1.

Supplementary Material 2. Supplementary Fig. 1. ACSLs expression comparisons in HCC patients collect from the TCGA-LIHC database. a mRNA expression of ACSLs between tumor samples (n=374) and normal samples (n=50). b ACSL4 protein was determined by western blotting in 18 representative pairs of HCC (TU) and adjacent non-tumor (NT) tissue samples, relative ACSL4 levels were normalized with α-tubulin or GAPDH. Supplementary Fig. 2. ACSL4 was associated with poorer HCC patient survival. a A Kaplan-Meier survival comparison of HCC patients with or without ACSL4 up-regulation. b With the increase of ACSL4 expression in tumor, the higher level of serum AFP observed. c-d Kaplan-Meier survival comparisons between ACSL4-present and ACSL4-absent HCC patients who had low serum AFP level (c) or who had high serum AFP level (d). e Accumulated risk to develop recurrence in HCC patients with or without ACSL4. f A Kaplan-Meier survival comparison of recurrent (within two years after curative surgery) HCC patients with or without ACSL4. g Kaplan-Meier survival comparison of recurrent HCC patients with different post-recurrent treatment methods.Supplementary Fig. 3. ACSL4 protected HCC cells from glucose starvation, rather than hypoxia or chemotherapeutic drugs. a Huh7 cells were silenced with ACSL4 siRNA or negative control siRNA, before treatment with full medium or glucose starvation for 48 hours. b ACSL4-overexpressing (OE) cells were treated with full medium or glucose starvation for 24 hours. The proteins were determined by Western blotting (up panel) while the proportion of survival cells were determined by PI assay (lower panel). c ACSL4-knockout (KO) Huh7 cells (left panel), ACSL4-OE N1S1 cells (middle panel) or ACSL4-OE HepG2 cells (right panel) were incubated in normoxia or hypoxia (Hpx) for indicated times. Then cell viability was determined by cck8 assay. d-e ACSL4-OE N1S1 cells (d) or ACSL4-OE HepG2 cells (e) were treated with cisplatin or doxorubicin for 36 hours or 24 hours respectively, with or without hypoxia treatment. f-i Comparation between NC-sg and ACSL4-KO Huh7 cells in cell proliferation (f), clone formation (g), migration (h) and invasion (i). j-m Comparation between NC and ACSL4-OE HepG2 cells in proliferation (j), clone formation (k), migration (l) and invasion (m). n Comparation between NC and ACSL4-OE N1S1 cells in cell proliferation. o Western blot analyses of ACSL4 in indicated cells incubated in full or glucose starvation medium for 48 hours. Compared with NC, #*P* <0.05,##*P* <0.01, ###*P*< 0.001. ***P <0.001. NS, not significant. Supplementary Fig. 4. ACSL4-OE maintenance of cellular ATP levels by promoting *β*-oxidation under glucose starvation. a-b ACSL4-OE N1S1 cells (a) or HepG2 cells (b) were treated with full medium or glucose starvation for 36 hours or 20 hours respectively. Then cellular ATP were detected. c ACSL4-OE cells were treated with glucose starvation for 12 hours, mitochondrial membrane potential was detected using JC-1 dye by flow cytometry. The ratios of red/green florescence were shown. d  Indicated cells treated with or without 50μM CQ were incubated in full or glucose starvation medium for 24 hours, then the cellular lipid droplets were staining with O oil red. e-f ACSL4-OE cells were treated with glucose starvation for 12 hours. The cellular lipid droplets (e) and triglycerides (f) were detected. g ACSL4-OE cells were treated with glucose starvation for 20 hours, then cellular *β*-hydroxybutyrate (*β*-HB) were detected. Supplementary Fig. 5. ACSL4 promote cell survival by mediating fatty acid activation and *β*-oxidation. a-bACSL4-OE N1S1 cells (a) or HepG2 cells (b) were treated with glucose starvation medium supplied with/without BSA or indicated BSA-fatty acid conjugates (AA-CoA, AA, LA, PA, OA, SA; 25 μM for each). Then the proportion of survival cells were detected. c-d ACSL4-OE N1S1 cells (upper panel) or HepG2 cells (lower panel) were treated with glucose starvation medium supplied with/without AA-CoA (25 μM), then cellular ATP (c) and *β*-HB (d) were detected respectively. e ACSL4-OE cells were treated with glucose starvation medium supplied with AA-CoA (25 μM) and/or etomoxir (ETO, 40 μM). The proportion of survival cells were detected. f-g Oxygen consumption rates (OCRs) in ACSL4-OE cells and respective control cells were determined by Seahorse XF analyzer (f). Basal OCR (left panel), ATP-linked OCR (middle panel) and maximal OCR (right panel) of indicated cells are summarized, respectively (g). Abbreviations: AA-CoA, arachidonoyl coenzyme A lithium salt; CON, control; BSA, bovine serum albumin; ETO, etomoxir; Oligo, oligomycin; Rot/ AA, Rotenone/ Antimycin A. Compared with NC, #*P* <0.05, ##*P* <0.01, ###*P* <0.001. *P<0.05, **P <0.01; ***P <0.001. NS, not significant. Supplementary Fig. 6. ACSL4-OE prevented cells from inhibition caused by canagliflozin mimicked glucose starvation *in vitro* and *in vivo*. a-b The 3D Spheroid tumor developed by indicated cells were treated with glucose starvation (a) or canagliflozin (Cana, 20 μM) in glucose-present (0.9 g/L) medium (b) for 60 hours. The dead cells were stained with PI (red fluorescence) while live cells were stained with calcein (green fluorescence) before observation under a fluorescent microscopy. The volume of spheroid (middle panel) and cell death (right panel) were detected. c The 3D Spheroid tumor developed by indicated cells were treated with canagliflozin (Cana, 20 μM) in glucose-present (0.9 g/L) medium in the present or absent of rosiglitazone (Rosig, 20 μM) for 48 hours. The volume of spheroid (lower-left panel) and cell death (lower-right panel) were detected. d-h The mice experiment was carried out as in Fig. 5c or S6i. The effects of Cana treatment on blood glucose level (d-e) and tumor glycogen (f) and body weights (g-h) were summarized respectively. h The mice bearing xenograft developed from ACSL4-OE or control HepG2 cells were treated with control PBS or Cana (30 mg/kg) for 15 days once every other day. The resected tumors were photographed (left panel). The tumor volumes (middle panel) and tumor weights (right panel) were measured. i-l Representative images of hematoxylin & eosin staining (HE), IHC for ACSL4 and Ki67 are shown (i). The proportion of area of necrotic tissue (k) and the Ki-67 positive score (l) were summarized. Compared with NC, #*P* <0.05, ##*P* <0.01, ###*P* <0.001. *P<0.05, ***P <0.001. NS, not significant. Supplementary Fig. 7. Correlation of CEBPA and ACSLs in HCC. a-d Correlation of mRNA expression between CEBPA and ACSLs (ACSL1, ACSL3, ACSL5, ACSL6) in HCC in TCGA-LIHC database. e-h Correlation of mRNA expression between CEBPA and ACSLs (ACSL1, ACSL3, ACSL5, ACSL6) in 6 human cell lines. i Expression of CEBPA protein in 7 cell lines. Supplementary Fig. 8. ACSL4 was regulated transcriptionally by CEBPA. a CEBPA-overexpression HepG2 (pcDNA3.1-CEBPA) and its negative control (pcDNA3.1-vector) were treated with glucose starvation for 24 hours. The proportion of survival cells is shown in the right panel.  b-c The luciferase reporter activity of ACSL4 promoter was assayed in indicated cells. Luciferase activity was normalized to Renilla activity and was plotted relative to NC (b) or pcDNA3.1-Vector (c). d Expression of CEBPA in different cancer types in TCGA database, which was analyzed using online website tool Timer 2.0 (http://timer.cistrome.org/), LIHC data was marked with the black frame. e-f Comparison of recurrence between ACSL4-High and ACSL4-Low HCC patients (e) or CEBPA-High and CEBPA-Low HCC patients (f) who treated TACE as post-recurrence treatment.

Supplementary Material 3.

## Data Availability

The RNA-seq data of TCGA-LIHC HCC patients used in this study are publicly available in Genomic Data Commons (https://portal.gdc.cancer.gov/). All other raw data are available upon request from the corresponding author.

## Abbreviations

AA: Arachidonic acid
AACoA: Arachidonoyl coenzyme A
AFP: Alpha-fetoprotein
ACSL4: Acyl-CoA synthetase long chain 4
Cana: Canagliflozin
CEBPA: CCAAT/enhancer binding protein α
CPT1: Carnitine palmitoyltransferase 1
EPO: Erythropoietin
*β*-HB: *β*-Hydroxybutyrate
HCC: Hepatocellular carcinoma
HIF-1α: Hypoxia inducible factor 1 alpha
OCR: Oxygen consumption rate
MMP: Mitochondrial membrane potential
NAFLD: Non-alcoholic fatty liver disease
PKM2,: Pyruvate kinase M2
SGLT2: Sodium-glucose transport protein 2
TACE: Transarterial chemo-embolization
TAE: Transarterial embolization
TASE: Transarterial Sorafenib-Embolization
TNBC: Triple-negative breast cancer

## References

[1] Sung H, Ferlay J, Siegel RL, Laversanne M, Soerjomataram I, Jemal A, et al. Global cancer statistics 2020: GLOBOCAN estimates of incidence and mortality worldwide for 36 cancers in 185 countries. CA Cancer J Clin. 2021;71(3):209–49. 33538338 10.3322/caac.21660

[2] Lu GD, Ang YH, Zhou J, Tamilarasi J, Yan B, Lim YC, et al. CCAAT/enhancer binding protein α predicts poorer prognosis and prevents energy starvation–induced cell death in hepatocellular carcinoma. Hepatology. 2015;61(3):965–78. 25363290 10.1002/hep.27593 PMC4365685

[3] Yang H, Deng Q, Ni T, liu Y, Lu L, Dai H, et al. Targeted Inhibition of LPL/FABP4/CPT1 fatty acid metabolic axis can effectively prevent the progression of nonalcoholic steatohepatitis to liver cancer. Int J Biol Sci. 2021;17(15):4207–22. 10.7150/ijbs.64714 PMC8579444 34803493

[4] Tang Y, Zhou J, Hooi SC, Jiang YM, Lu GD. Fatty acid activation in carcinogenesis and cancer development: Essential roles of long-chain acyl-CoA synthetases (Review). Oncol Lett. 2018;16(2):1390–6. 30008815 10.3892/ol.2018.8843 PMC6036470

[5] Chen J, Ding C, Chen Y, Hu W, Yu C, Peng C, et al. ACSL4 reprograms fatty acid metabolism in hepatocellular carcinoma via c-Myc/SREBP1 pathway. Cancer Lett. 2021;502:154–65. 33340617 10.1016/j.canlet.2020.12.019

[6] Feng J, Lu PZ, Zhu GZ, Hooi SC, Wu Y, Huang XW, et al. ACSL4 is a predictive biomarker of sorafenib sensitivity in hepatocellular carcinoma. Acta Pharmacol Sin. 2021;42(1):160–70. 32541921 10.1038/s41401-020-0439-x PMC7921679

[7] Reig M, Forner A, Rimola J, Ferrer-Fàbrega J, Burrel M, Garcia-Criado Á, et al. BCLC strategy for prognosis prediction and treatment recommendation: The 2022 update. J Hepatol. 2022;76(3):681–93. 34801630 10.1016/j.jhep.2021.11.018 PMC8866082

[8] Lencioni R, Kudo M, Ye SL, Bronowicki JP, Chen XP, Dagher L, et al. GIDEON (Global Investigation of therapeutic DEcisions in hepatocellular carcinoma and Of its treatment with sorafeNib): Second interim analysis. Int J Clin Pract. 2014;68(5):609–17. 24283303 10.1111/ijcp.12352 PMC4265239

[9] Wang Z, Ren Z, Chen Y, Hu J, Yang G, Yu L, et al. Adjuvant transarterial chemoembolization for HBV-related hepatocellular carcinoma after resection: A randomized controlled study. Clin Cancer Res. 2018;24(9):2074–81. 29420221 10.1158/1078-0432.CCR-17-2899

[10] Wei W, Jian P, Li S, Guo Z, Zhang Y, Ling Y, et al. Adjuvant transcatheter arterial chemoembolization after curative resection for hepatocellular carcinoma patients with solitary tumor and microvascular invasion: a randomized clinical trial of efficacy and safety. Cancer Commun (Lond). 2018;38(1):61. 30305149 10.1186/s40880-018-0331-y PMC6235393

[11] He Y, Qian J, Zhu G, Wu Z, Cui L, Tu S, et al. Development and validation of nomograms to evaluate the survival outcome of HCC patients undergoing selective postoperative adjuvant TACE. Radiol Med. 2024;129(4):653–64. 38512609 10.1007/s11547-024-01792-0

[12] Zhong C, Niu Y, Liu W, Yuan Y, Li K, Shi Y, et al. S100A9 Derived from Chemoembolization-Induced Hypoxia Governs Mitochondrial Function in Hepatocellular Carcinoma Progression. Adv Sci. 2022;9(30):2202206. 10.1002/advs.202202206 PMC9596847 36041055

[13] Rashid M, Zadeh LR, Baradaran B, Molavi O, Ghesmati Z, Sabzichi M, et al. Up-down regulation of HIF-1α in cancer progression. Gene. 2021;798: 145796. 34175393 10.1016/j.gene.2021.145796

[14] Meyer T, Kirkwood A, Roughton M, Beare S, Tsochatzis E, Yu D, et al. A randomised phase II/III trial of 3-weekly cisplatin-based sequential transarterial chemoembolisation vs embolisation alone for hepatocellular carcinoma. Br J Cancer. 2013;108(6):1252–9. 23449352 10.1038/bjc.2013.85 PMC3619271

[15] Brown KT, Do RK, Gonen M, Covey AM, Getrajdman GI, Sofocleous CT, et al. Randomized trial of hepatic artery embolization for hepatocellular carcinoma using doxorubicin-eluting microspheres compared with embolization with microspheres alone. J Clin Oncol. 2016;34(17):2046–53. 26834067 10.1200/JCO.2015.64.0821 PMC4966514

[16] Li YJ, Fahrmann JF, Aftabizadeh M, Zhao Q, Tripathi SC, Zhang C, et al. Fatty acid oxidation protects cancer cells from apoptosis by increasing mitochondrial membrane lipids. Cell Rep. 2022;39(9): 110870. 35649368 10.1016/j.celrep.2022.110870

[17] Neal B, Perkovic V, Mahaffey KW, de Zeeuw D, Fulcher G, Erondu N, et al. Canagliflozin and Cardiovascular and Renal Events in Type 2 Diabetes. N Engl J Med. 2017;377(7):644–57. 28605608 10.1056/NEJMoa1611925

[18] Calle EE, Rodriguez C, Walker-Thurmond K, Thun MJ. Overweight, obesity, and mortality from cancer in a prospectively studied cohort of U.S. Adults. N Engl J Med. 2003;348(17):1625–38. 12711737 10.1056/NEJMoa021423

[19] Duan J, Wang Z, Duan R, Yang C, Zhao R, Feng Q, et al. Therapeutic targeting of hepatic ACSL4 ameliorates non-alcoholic steatohepatitis in mice. Hepatology. 2021;75(1):140–53. 34510514 10.1002/hep.32148 PMC8688219

[20] Sánchez-Martínez R, Cruz-Gil S, García-Álvarez MS, Reglero G, de Ramírez Molina A. Complementary ACSL isoforms contribute to a non-Warburg advantageous energetic status characterizing invasive colon cancer cells. Sci Rep. 2017;7(1):11143. 28894242 10.1038/s41598-017-11612-3 PMC5593891

[21] Wu X, Li Y, Wang J, Wen X, Marcus M, Daniels G, et al. Long chain fatty Acyl-CoA synthetase 4 is a biomarker for and mediator of hormone resistance in human breast cancer. PLoS ONE. 2013;8(10): e77060. 24155918 10.1371/journal.pone.0077060 PMC3796543

[22] Wu D, Zuo Z, Sun X, Li X, Yin F, Yin W. ACSL4 promotes malignant progression of Hepatocellular carcinoma by targeting PAK2 transcription. Biochem Pharmacol. 2024;224: 116206. 38615921 10.1016/j.bcp.2024.116206

[23] Junttila MR, de Sauvage FJ. Influence of tumour micro-environment heterogeneity on therapeutic response. Nature. 2013;501(7467):346–54. 24048067 10.1038/nature12626

[24] Quail DF, Joyce JA. Microenvironmental regulation of tumor progression and metastasis. Nat Med. 2013;19(11):1423–37. 24202395 10.1038/nm.3394 PMC3954707

[25] Chen J, Ding C, Chen Y, Hu W, Lu Y, Wu W, et al. ACSL4 promotes hepatocellular carcinoma progression via c-Myc stability mediated by ERK/FBW7/c-Myc axis. Oncogenesis. 2020;9(4):42. 32350243 10.1038/s41389-020-0226-z PMC7190855

[26] Lencioni R, de Baere T, Soulen M, Rilling W, Geschwind J. Lipiodol transarterial chemoembolization for hepatocellular carcinoma: A systematic review of efficacy and safety data. Hepatology. 2016;64(1):106–16. 26765068 10.1002/hep.28453

[27] Cappelli A, Cucchetti A, Cabibbo G, Mosconi C, Maida M, Attardo S, et al. Refining prognosis after trans-arterial chemo-embolization for hepatocellular carcinoma. Liver Int. 2016;36(5):729–36. 26604044 10.1111/liv.13029

[28] Kadalayil L, Benini R, Pallan L, O’Beirne J, Marelli L, Yu D, et al. A simple prognostic scoring system for patients receiving transarterial embolisation for hepatocellular cancer. Ann Oncol. 2013;24(10):2565–70. 23857958 10.1093/annonc/mdt247 PMC4023407

[29] Kim BK, Shim JH, Kim SU, Park JY, Kim DY, Ahn SH, et al. Risk prediction for patients with hepatocellular carcinoma undergoing chemoembolization: development of a prediction model. Liver Int. 2016;36(1):92–9. 25950442 10.1111/liv.12865

[30] Wei X, Zhao L, Ren R, Ji F, Xue S, Zhang J, et al. MiR-125b loss activated HIF1α/pAKT loop, leading to Transarterial chemoembolization eesistance in hepatocellular carcinoma. Hepatology. 2021;73(4):1381–98. 32609900 10.1002/hep.31448 PMC9258000

[31] Wang JH, Zhong XP, Zhang YF, Wu XL, Li SH, Jian PE, et al. Cezanne predicts progression and adjuvant TACE response in hepatocellular carcinoma. Cell Death Dis. 2017;8(9): e3043. 28880268 10.1038/cddis.2017.428 PMC5636974

[32] Gai X, Zhou P, Xu M, Liu Z, Zheng X, Liu Q. Hyperactivation of IL-6/STAT3 pathway leaded to the poor prognosis of post-TACE HCCs by HIF-1α/SNAI1 axis-induced epithelial to mesenchymal transition. J Cancer. 2020;11(3):570–82. 31942180 10.7150/jca.35631 PMC6959052

[33] Lin ZH, Jiang JR, Ma XK, Chen J, Li HP, Li X, et al. Prognostic value of serum HIF-1α change following transarterial chemoembolization in hepatocellular carcinoma. Clin Exp Med. 2021;21(1):109–20. 33037574 10.1007/s10238-020-00667-8

[34] Martin SP, Fako V, Dang H, Dominguez DA, Khatib S, Ma L, et al. PKM2 inhibition may reverse therapeutic resistance to transarterial chemoembolization in hepatocellular carcinoma. J Exp Clin Cancer Res. 2020;39(1):99. 32487192 10.1186/s13046-020-01605-y PMC7268641

[35] Zhou J, Feng J, Wu Y, Dai HQ, Zhu GZ, Chen PH, et al. Simultaneous treatment with sorafenib and glucose restriction inhibits hepatocellular carcinoma in vitro and in vivo by impairing SIAH1-mediated mitophagy. Exp Mol Med. 2022;54(11):2007–21. 36385558 10.1038/s12276-022-00878-x PMC9723179

[36] Li B, Li Y, Zhou H, Xu Y, Cao Y, Cheng C, et al. Multiomics identifies metabolic subtypes based on fatty acid degradation allocating personalized treatment in hepatocellular carcinoma. Hepatology. 2024;79(2):289–306. 37540187 10.1097/HEP.0000000000000553 PMC10789383

[37] Rambold Angelika S, Cohen S, Lippincott-Schwartz J. Fatty Acid Trafficking in Starved Cells: Regulation by Lipid Droplet Lipolysis, Autophagy, and Mitochondrial Fusion Dynamics. Dev Cell. 2015;32(6):678–92. 25752962 10.1016/j.devcel.2015.01.029 PMC4375018

[38] Nguyen TB, Louie SM, Daniele JR, Tran Q, Dillin A, Zoncu R, et al. DGAT1-Dependent Lipid Droplet Biogenesis Protects Mitochondrial Function during Starvation-Induced Autophagy. Dev Cell. 2017;42(1):9-21.e5. 28697336 10.1016/j.devcel.2017.06.003 PMC5553613

[39] Zhou J, Yu L, Gao X, Hu J, Wang J, Dai Z, et al. Plasma MicroRNA Panel to Diagnose Hepatitis B Virus-Related Hepatocellular Carcinoma. J Clin Oncol. 2011;29(36):4781–8. 22105822 10.1200/JCO.2011.38.2697

[40] Qin X, Zhang J, Lin Y, Sun XM, Zhang JN, Cheng ZQ. Identification of MiR-211-5p as a tumor suppressor by targeting ACSL4 in Hepatocellular Carcinoma. J Transl Med. 2020;18(1):326. 32859232 10.1186/s12967-020-02494-7 PMC7456023

[41] Yao X, Hu J-F, Daniels M, Yien H, Lu H, Sharan H, et al. A Novel Orthotopic Tumor Model to Study Growth Factors and Oncogenes in Hepatocarcinogenesis1. Clin Cancer Res. 2003;9(7):2719–26. 12855652

[42] Tischfield D, Gurevich A, Johnson O, Gatmaytan I, Nadolski G, Soulen M, et al. Transarterial Embolization Modulates the Immune Response within Target and Nontarget Hepatocellular Carcinomas in a Rat Model. Radiology. 2022;303(1):215–25. 35014906 10.1148/radiol.211028 PMC8962821

