# Supplementary material for: The prognostic role of ACSL4 in postoperative adjuvant TACE-treated HCC: implications for therapeutic response and mechanistic insights
Source: J Exp Clin Cancer Res. 2024 Nov 19;43:306. doi: 10.1186/s13046-024-03222-5 (PMC11575417; doi:10.1186/s13046-024-03222-5)
Supplement: Supplementary file 1 — Supplementary Material 1. [file 13046_2024_3222_MOESM1_ESM.docx]

**Supplementary Material and Methods**

1. ***Bioinformatic analysis***

The mRNA transcriptome profiling data (HTSeq - FPKM) were downloaded from TCGA-LIHC database (https://portal.gdc.cancer.gov/). The data were proceeded using RStudio with “limma” package. Wilcox’s test was used to assess the differential expression of target genes between tumor and normal samples. Correlation of mRNA expression between CEBPA and ACSL4 in HCC was analyzed using Timer 2.0 online database (http://timer.cistrome.org/).

1. ***Cell Culture and Treatment***

Human Huh7, Hep3B, HepG2 cell lines were purchased from the National Collection of Authenticated Cell Cultures (Shanghai, China), PLC/PRF/5 (PLC/5), SNU-398 cell lines and rat N1S1 cell lines were purchased from American Type Culture Collection (Manassas, VA, USA), while HCC-M was kindly provided by Dr. Ren EC (National University of Singapore). Cell lines were cultured in DMEM medium or RPMI-1640 medium (SNU-398) or IMDM medium (N1S1) respectively, supplemented with 10% FBS (Gibco, Newcastle, NSW, Australia). Cells were washed with PBS twice before nutrient starvation in DMEM medium with or without glucose (4.5 g/L) and/or glutamine (4.0 mM) [1]. Cell survival was determined with propidium iodide (PI, #P4170, Sigma-Aldrich, Shanghai, China) exclusion assay in a flow cytometer (CytoFLEX FCM, Beckman, Atlanta, USA) as previously [2].

1. ***Statistical analyses***

The experimental differences were analyzed with the SPSS software package (version 22.0) using one-way ANOVA analysis, Kruskal-Wallis test, two-sided or paired Student’s *t* test, *Chi*-Square test or Fisher’s exact test analysis. Correlation of indicated variables were analyzed using Spearman's rank correlation or Pearson’s rank correlation. Survival analyses of HCC patients were performed using Kaplan-Meier and multivariate Cox regression models as described previously [3]. Quantitative data are expressed as the mean ± SD of at least three independent experiments. Statistical significance was set at *P* < 0.05.

1. ***Western Immunoblotting (WB) and Immunochemistry (IHC)***

Western Immunoblotting and IHC were carried out using standard techniques. Antibodies used in this study were listed in SMM-table 1.

SMM-table 1. Antibodies.

| **Antibodies** | **Source** | **Identifier** | **Application** |
| --- | --- | --- | --- |
| ACSS2 | Santa Cruz Biotechnology | #sc-398559 | 1:1000 for WB |
| ACSL3 | Santa Cruz Biotechnology | #sc-271246 | 1:1000 for WB |
| ACSL4 | Santa Cruz Biotechnology | # sc-271800 | 1:100 for IHC (Singapore cohort) |
| α-tubulin | Sigma-Aldrich | #T5168 | 1:10000 for WB |
| ACSL1 | Cell Signaling Technology | #9189 | 1:1000 for WB |
| CEBPA | Cell Signaling Technology | #2295 | 1:1000 for WB; 1:100 for IHC |
| ACSL4 | Abcam | #ab155282 | 1:1000 for WB; 1:250 for IHC (Guangxi cohort) |
| Ki-67 | Abcam | #ab92742 | 1:250 for IHC |
| GAPDH | Affinity | # AF7021 | 1:2000 for WB |

1. ***Cell proliferation and toxicity assay***

For cell proliferation assay, cells were seed at an initial density of 2000 cells per well in a 96-well plate, incubated for 1-5 or 6 days. For cell toxicity assay, cells were seed at an initial density of 6000-8000 cells per well in a 96-well plate, then treated with indicated chemical compounds for 24-48 hours. Cell proliferation or viability was determined by Cell Counting Kit-8 assay (#HY-K0301, MedChemExpress, Shanghai, China) according to the manufacturer's instructions.

1. ***Clone formation assay***

HCC cells were seed at an initial density of 1000 cells per well in a 6-well plate, incubated for 12 days. Then cells were wash twice with 1×PBS and fixed with 1 mL paraformaldehyde for 20 min; Remove paraformaldehyde, add 1 mL of 0.5% crystal violet solution to each well and stain at room temperature for 20 min, remove the staining solution, gently rinse the residual staining solution with water, dry them at room temperature and scan them with Expression 11000XL scanner (Epson, Shanghai, China). Clone numbers were counted using ImageJ software.

1. ***Wound healing migration assay***

Draw a straight line in the confluent cells using a 1000 μL tip, wash twice with PBS, then add 1% FBS medium to each well, wound pictures were recorded at 0, 48 hours, respectively. The width of wound was measured using ImageJ software. Wound area (%) was calculated using the formula (100 × wound width at 48 hours / wound width at 0 hour).

1. ***Invasion assay***

The invasion assay was conducted in 24-well Transwell insert (#TCS003024, JET biofil, Guangzhou, China). 5×10^4^ HCC cells were seed at Matrigel-coated upper chamber. Put upper chamber into lower chamber filled with 20% FBS medium, incubated for 48 hours. Then cells were fixed with 4% paraformaldehyde for 20 min and stained with 0.1% crystal violet for 15 min. The cells were counted in five random field using ImageJ software.

1. ***Nile red staining***

For nile red staining, cells were stained with 1 μM nile red working solution (#72485, Sigma-Aldrich) at 37℃ in the dark for 30 minutes, then cells were washed twice with PBS. 5×10^4^ cells were harvested by centrifugation (1000 × g for 3 minutes) and resuspended in 500 μL FBS-free DMEM before the flow cytometry analysis (fluorescence was detected using PE-A channel).

1. ***Mitochondrial membrane potential (ΔΨm) assay***

Cells were seeded at an initial density of 50,000 cells per well in a 12-well plate for 24 hours. Then, cells were incubated with full medium or glucose-deprived medium for 12 hours. After incubation, mitochondrial ΔΨm of cells were detected with Mitochondrial Membrane Potential Assay Kit with JC-1 (#M8650, Solrabio, Beijing, China) in accordance with the manufacturer's instructions. In brief, cells were washed twice with phosphate buffered solution (PBS) and incubated with 1× JC-1 working solution at 37℃ in the dark for 20 minutes. Then, cells were washed twice with 1× JC-1 buffer and harvested in 1× JC-1 buffer for mitochondrial ΔΨm assay by flow cytometry. Mean of fluorescence intensity (MFI) of JC1 red/ JC1 green were analyzed using FlowJo (Tree Star Technologies) and the lower ratio of JC-1 red/ JC-1 green represented the decreased mitochondrial ΔΨm.

1. ***RNAi and Gene Overexpression***

The construction of ACSL4 CRISPR/Cas9 knockout plasmids and establishment of stable cells were performed as described previously [4]. The sequences for single guide RNA (sgRNA, Supplementary Methods) were designed using CHOPCHOP guide RNA design websites (http://chopchop.cbu.uib.no) and synthesized by Sangon Biotech (Shanghai, China). After transfection using Lipofectamine™ 3000 (Thermo Fisher), cells were cultured in selective medium containing 0.5 μg/mL puromycin (Thermo Fisher). In addition, Huh7 cells were transfected with negative control or CEBPA CRISPR/Cas9 knockout lentivirus (Genechem, Shanghai, China). For transient knockdown, siRNAs against negative control (#12935300), ACSL4 (#HSS103537, #HSS103539) from Thermo Fisher were given to cells with the aid of Lipofectamine RNAiMAX (Thermo Fisher). ACSL4 over-expression experiment with ready-made lentivirus (GeneChem) were conducted in HepG2 cells and N1S1 cells according to the manufacturer’s instructions. HepG2 cells were transfected with pcDNA3.1 vector or pcDNA3.1-CEBPA plasmids kindly provided by Dr. A.D. Friedman (Johns Hopkins University, Baltimore, MD) [5].

**SgRNA sequences**

| Gene | top sequences (5’ to 3’) | bottom sequence (5’ to 3’) |
| --- | --- | --- |
| ACSL4-sg1 | GTCCGCATGATGCTGTCTGGAGG | CCTCCAGACAGCATCATGCGGAC |
| ACSL4-sg2 | GCATCATCACTCCCTTAGGTCGG | CCGACCTAAGGGAGTGATGATGC |
| Negative control (NC) | CGCTTCCGCGGCCCGTTCAA | TTGAACGGGCCGCGGAAGCG |
| CEBPA-sg1 | GCCCCGACGCGCTCGTACAG | CTGTACGAGCGCGTCGGGGC |
| CEBPA-sg2 | TTGGCTTCATCCTCCTCGCG | CGCGAGGAGGATGAAGCCAA |

1. ***Seahorse Oxygen Consumption Assay and Fatty Acid Oxidation Stress Assay***

The mitochondrial oxygen consumption rates (OCR) and fatty acid oxidation (FAO) levels were determined by a Seahorse XF24 analyzer (Agilent Technologies) as previously [3] or according to the manufacturer’s instruction. For FAO experiment, briefly 4×10^4^ ACSL4-knockout Huh7 cells or ACSL4-overexpressed HepG2 cells and corresponding control cells were sub-cultured into XF24 cell culture microplates (Agilent Technologies) overnight, before exchange of medium to FAO assay buffer (non-buffered XF DMEM, Agilent Technologies) supplemented with 2 mM glucose and 0.5 mM L-carnitine. The final working concentrations for each drug used for FAO assays were: BSA-conjugated arachidonic acids (AA 25 μM), etomoxir (ETO 40 μM, from MedChemExpress), oligomycin (Oligo 1.5 μM, from Agilent), FCCP (0.5 μM) and rotenone/antimycin A (Rot/AA, 0.5 μM for each).

1. ***Tumor 3D Spheroid Models***

ACSL4 genetic modified HCC cells were seeded into 96-well U-shaped-bottom Nunclon Sphera Microplates (Thermo Fisher) to form spheroids, before treatment with 20 μM canagliflozin in DMEM (0.9 g/L glucose). Cell viability was tested using Calcein/PI Cell Viability/Cytotoxicity Assay Kit (Beyotime). Fluorescent images of Calcein (green, for live cells) /PI (red, for dead cells) stained spheroids were captured using EVOS™ FL Auto Imaging System (Thermo Fisher) as before [1].

1. ***Ectopic HCC Xenograft Experiments***

All animal experiments were approved by the Guangxi Medical University Institutional Animal Care and Use Committees (#201910029) and then performed in accordance with the Association for Assessment and Accreditation of Laboratory Animal Care guidelines. Male BALB/c nude mice (6-8 weeks old) from Hunan SJA Laboratory Animal Co., Ltd. (Changsha, China) were housed in an SPF laboratory with free access to water and food. For ACSL4-knockout xenograft experiment, a total of 32 mice were randomly divided into four groups (8 for each), and then inoculated with 1×10^6^ Huh7-NC cells or ACSL4-sg2 Huh7 cells subcutaneously into the right flank. For ACSL4-overexpressing xenograft experiment, a total of 36 mice were randomly divided into four groups (9 for each), and then inoculated with 1×10^6^ HepG2-NC cells or ACSL4-OE HepG2 cells. Once the inoculated tumor size reached 50-100 mm^3^, the mice were given PBS or canagliflozin treatment (30 mg/kg, once every other day) by gavage. Tumors were measured every day with digital calipers, and tumor volume was calculated using formula (length × width × width)/2. Blood glucose was measured before gavage treatment and after 18 hours of treatment using an ACCU CHEK glucose meter (Roche). After mice were sacrificed, tumors weights and size were measured. Then tumors were collected for subsequent experiments. The IHC experiments were carried out as before [1].

1. ***Glycogen assay***

Glycogen in the tumor samples collected from animal experiments were detected using Glycogen Assay Kit (#E-BC-F040, Elabscience, Wuhan, China). Briefly, every tumor sample (10mg) was homogenized with 90 µL ddH_2_O for 10 minutes on ice. Boil the homogenates at 95 ℃ for 10 minutes. Centrifuge at 12,000 × g for 10 minutes at 4 ℃ and collect the supernatant. Diluted supernatants (1:100) were assayed according to the manufacturer’s instructions.

1. ***Oil red O assay***

Cells were seed at a density of 2×10^5^ cells per well at 6-well plate for 24 hours. Then, cells were treated with full medium or glucose deprived medium for indicated hours before staining with oil red O staining solution (#C1058S, Beyotime, Shanghai, China) according the manufacturer’s instructions. Briefly, cells were washed with PBS twice. Add 4% paraformaldehyde fixative solution for 10 minutes and rinse with PBS twice. Add 1 mL washing solution to cover the cells for 20 seconds. Absorb the washing solution, add 1 mL oil red O staining solution, and dye for 20 minutes. Remove the oil red O staining solution, add 1 mL washing solution for 30 seconds, then remove the washing solution. Cover the cells evenly with 1 mL PBS for pictures captured.

1. ***Quantitative real-time PCR (RT-qPCR)***

Total RNA was extracted from nontumor or tumor tissues by RNAiso Plus (#9019, Takara, Beijing, China) according to manufacturer’s instruction. After cDNA synthesis using PrimeScript^TM^ RT Master Mix (#RR036A, Takara), samples were analyzed using pairs of primers specific for indicated genes. Sequence-specific amplification was detected by TB Green® Premix EX TaqTM II (#RR820A, Takara) by using StepOnePlus^TM^ Real-Time PCR system (Thermo Fisher Scientific). The human SFRS4 or ACTB was used as an internal control to normalize target gene mRNA levels. Primers were ordered from Sangon Biotech (Shanghai, China) and summarized in the SMM-table 2.

SMM-table 2.

| **Gene** | **Forward primer sequence (5’ to 3’)** | **Reverse primer sequence (5’ to 3’)** |
| --- | --- | --- |
| ACSL1 | GCGACCTCTCCATGCAGTC | CAAGTAGTGCGGATCTTCGTG |
| ACSL3 | TCAAACCGAATTAAAGCAAAGCC | TGAAGCCAAACCATCCAAACTAT |
| ACSL4 | AACCCAGAAAACTTGGGCATT | GTCGGCCAGTAGAACCACT |
| ACSL5 | TGGATCATCTCCGAATTGGCT | AGGTGTCATACAGAGGTACAGC |
| ACSL6 | GCACGGCGATCTGTGATTG | GGCGGAACACCTGGTACAT |
| ACSS1 | CACAGGACAGACAACAAGGTC | CCTGGGTATGGACGATGCC |
| ACSS2 | CAAGTGTGTCAGTTCAGCAATG | CCACAAGCTCTGGGATCATAGG |
| ACSS3 | TGGACCAAAACGCTGGAGAAC | ACGATCAACGGCATTGTAACA |
| CEBPA | TGTATACCCCTGGTGGGAGA | TCATAACTCCGGTCCCTCTG |
| ACTB | GGACTTCGAGCAAGAGATGG | AGCACTGTGTTGGCGTACAG |
| SFRS4 | GCCCTCCTACTCGCACAGA | GCGTCCCTTGTGAGCATCT |
| ACSL4 promoter | GGGAAAATGGAAATCAGCAAGT | GCGAACTATCTGAGGGCTTCAA |

1. ***ChIP-qPCR***

ChIP assay was performed using BeyoChIPTM ChIP assay kit (#P2080, Beyotime) and following the manufacturer’s instructions. Briefly, RNA from indicated cells was isolated and fragmented by sonication for 10 seconds with 5 seconds intervals for 24 cycles at 0°C. Keep a portion of the lysate as input sample, Protein A/G magnetic beads were incubated with anti-CEBPA antibody (#8176, Cell signaling technology, 1:50 for ChIP) or normal rabbit IgG antibody (#2729, Cell signaling technology, 1:50 for ChIP) for 16 hours at 4°C, Then, the anti-CEBPA/ IgG antibody-bound magnetic beads were washed, added to elution buffer (1% SDS, 0.1 M NaHCO_3_) and incubated for 15 minutes at 65°C. Then eluent and beads were separated by using DynaMag™-2 (#12321D, Thermo fisher). The purified DNA samples were analyzed by RT-qPCR, and the PCR products were detected by agarose gel electrophoresis. The primers of ACSL4 promoter region were listed in SMM-table 2.

1. ***Dual-luciferase reporter assay***

Putative motifs of ACSL4 promoter binding with CEBPA predicted by JASPAR database (https://jaspar.elixir.no/). A variety of different length ACSL4 promoter fragments (-2000 ~ +1, -1670 ~ +1, -1520 ~ +1, -1340 ~ +1, -1100 ~ +1, -850 ~ +1, -450 ~ +1) or CEBPA-binding motif-deleted mutation fragments (motif 1-deleted, motif 3-deleted, motif 7-deleted, motif 1+3-deleted, motif 1+7-deleted, motif 3+7-deleted and motif 1+3+7-deleted) were synthesized by Hunan Fenghui Biotechnology Co., Ltd (Hunan, China) and cloned into the pGL3-Basic Vector (#E1751, Promega, USA) to construct luciferase reporter plasmid. The indicated cells were co-transfected with luciferase reporter plasmid and pRL-TK (Renilla) plasmid (#E2241, Promega) for 24 hours. Then the ACSL4 promoter activity of cells was assays using Dual-Luciferase Reporter Assay System (#E1910, Promega, USA) following the manufacturer’s instructions.

1. **List of chemical reagents and assay kits**

Chemical reagents and assay kits used in this study were summarized in the SMM-table 3.

SMM-table 3. Chemical reagents and assay kits.

| **Item** | **Source** | **Identifier** |
| --- | --- | --- |
| **Chemical Reagents** |  |  |
| Triacsin C | Sigma-Aldrich | #T4540 |
| Rosiglitazone | Sigma-Aldrich | #R2408 |
| Cisplatin | MedChemExpress | #HY-17394 |
| Doxorubicin | MedChemExpress | #HY-15142A |
| Etomoxir | MedChemExpress | #HY-50202 |
| Bovine serum albumin (fatty acid-free) | Solarbio | #A8850 |
| Arachidonic acid | Aladdin | #A131025 |
| Linoleic acid | Aladdin | #L100447 |
| Oleic acid | Aladdin | #O108487 |
| Palmitic acid | Aladdin | #P298771 |
| Stearic acid | Aladdin | #S298767 |
| Arachidonoyl coenzyme A lithium salt | Sigma-Aldrich | #A5837 |
| Nile red | Sigma-Aldrich | #72485 |
| Propidium iodide | Sigma-Aldrich | #P4170 |
| Lipofectamine RNAiMAX | Thermo Fisher | #13778150 |
| Lipofectamine 3000 | Thermo Fisher | # L3000015 |
| Matrigel  Continued to SMM-table 3. | Corning | #354234 |
| **Item** | **Source** | **Identifier** |
| **Reagent kits** |  |  |
| Oil red O staining solution | Beyotime | #C1058S |
| Enhanced ATP assay kit | Beyotime | #S0027 |
| Calcein/PI cell viability/ cytotoxicity assay kit | Beyotime | #C2015M |
| TUNEL assay kit | Beyotime | #C1090 |
| BeyoChIP^TM^ ChIP assay kit | Beyotime | #P2080 |
| DNA purification kit | Beyotime | #D0033 |
| β-Hydroxybutyrate colorimetric assay kit | Cayman Chemical | #700190 |
| PicoProbeTM triglyceride (TG) fluorometric assay kit | Abcam | #K614 |
| Seahorse XF cell mito stress test kit | Agilent Technologies | #103015-100 |
| Glycogen assay kit | Elabscience | #E-BC-F040 |
| Dual-Luciferase reporter assay system | Promega | #E1910 |

**References**

1. Zhou J, Feng J, Wu Y, Dai HQ, Zhu GZ, Chen PH, et al. Simultaneous treatment with sorafenib and glucose restriction inhibits hepatocellular carcinoma in vitro and in vivo by impairing SIAH1-mediated mitophagy. Exp Mol Med. 2022;54(11):2007-21.

2. Zhou J, Tan SH, Nicolas V, Bauvy C, Yang ND, Zhang J, et al. Activation of lysosomal function in the course of autophagy via mTORC1 suppression and autophagosome-lysosome fusion. Cell Res. 2013;23(4):508-23.

3. Lu GD, Ang YH, Zhou J, Tamilarasi J, Yan B, Lim YC, et al. CCAAT/enhancer binding protein α predicts poorer prognosis and prevents energy starvation–induced cell death in hepatocellular carcinoma. Hepatology. 2015;61(3):965-78.

4. Lu G, Tan HWS, Schmauck-Medina T, Wang L, Chen J, Cho YL, et al. WIPI2 positively regulates mitophagy by promoting mitochondrial recruitment of VCP. Autophagy. 2022;18(12):2865-79.

5. Paz-Priel I, Ghosal AK, Kowalski J, Friedman AD. C/EBPα or C/EBPα oncoproteins regulate the intrinsic and extrinsic apoptotic pathways by direct interaction with NF-κB p50 bound to the bcl-2 and FLIP gene promoters. Leukemia. 2009;23(2):365-74.
