# Supplementary material for: The prognostic role of ACSL4 in postoperative adjuvant TACE-treated HCC: implications for therapeutic response and mechanistic insights
Source: J Exp Clin Cancer Res. 2024 Nov 19;43:306. doi: 10.1186/s13046-024-03222-5 (PMC11575417; doi:10.1186/s13046-024-03222-5)
Supplement: Supplementary file 2 — Supplementary Material 2. Supplementary Fig. 1. ACSLs expression comparisons in HCC patients collect from the TCGA-LIHC database. a mRNA expression of ACSLs between tumor samples (n=374) and normal samples (n=50). b ACSL4 protein was determined by western blotting in 18 representative pairs of HCC (TU) and adjacent non-tumor (NT) tissue samples, relative ACSL4 levels were normalized with α-tubulin or GAPDH. Supplementary Fig. 2. ACSL4 was associated with poorer HCC patient survival. a A Kaplan-Meier survival comparison of HCC patients with or without ACSL4 up-regulation. b With the increase of ACSL4 expression in tumor, the higher level of serum AFP observed. c-d Kaplan-Meier survival comparisons between ACSL4-present and ACSL4-absent HCC patients who had low serum AFP level (c) or who had high serum AFP level (d). e Accumulated risk to develop recurrence in HCC patients with or without ACSL4. f A Kaplan-Meier survival comparison of recurrent (within two years after curative surgery) HCC patients with or without ACSL4. g Kaplan-Meier survival comparison of recurrent HCC patients with different post-recurrent treatment methods.Supplementary Fig. 3. ACSL4 protected HCC cells from glucose starvation, rather than hypoxia or chemotherapeutic drugs. a Huh7 cells were silenced with ACSL4 siRNA or negative control siRNA, before treatment with full medium or glucose starvation for 48 hours. b ACSL4-overexpressing (OE) cells were treated with full medium or glucose starvation for 24 hours. The proteins were determined by Western blotting (up panel) while the proportion of survival cells were determined by PI assay (lower panel). c ACSL4-knockout (KO) Huh7 cells (left panel), ACSL4-OE N1S1 cells (middle panel) or ACSL4-OE HepG2 cells (right panel) were incubated in normoxia or hypoxia (Hpx) for indicated times. Then cell viability was determined by cck8 assay. d-e ACSL4-OE N1S1 cells (d) or ACSL4-OE HepG2 cells (e) were treated with cisplatin or doxorubicin for 36 hours or 24 [file 13046_2024_3222_MOESM2_ESM.pdf]

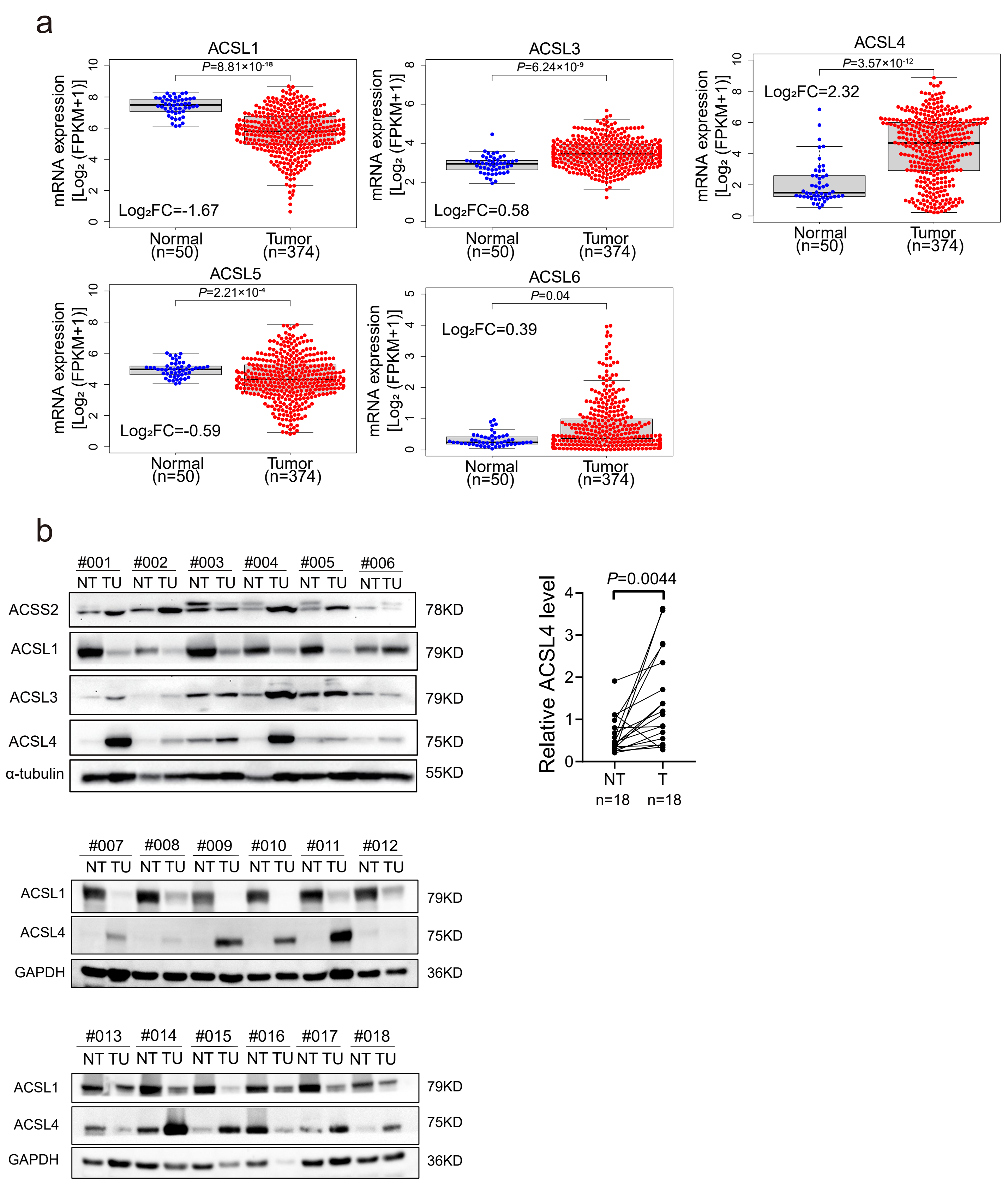

Supplementary figure 1

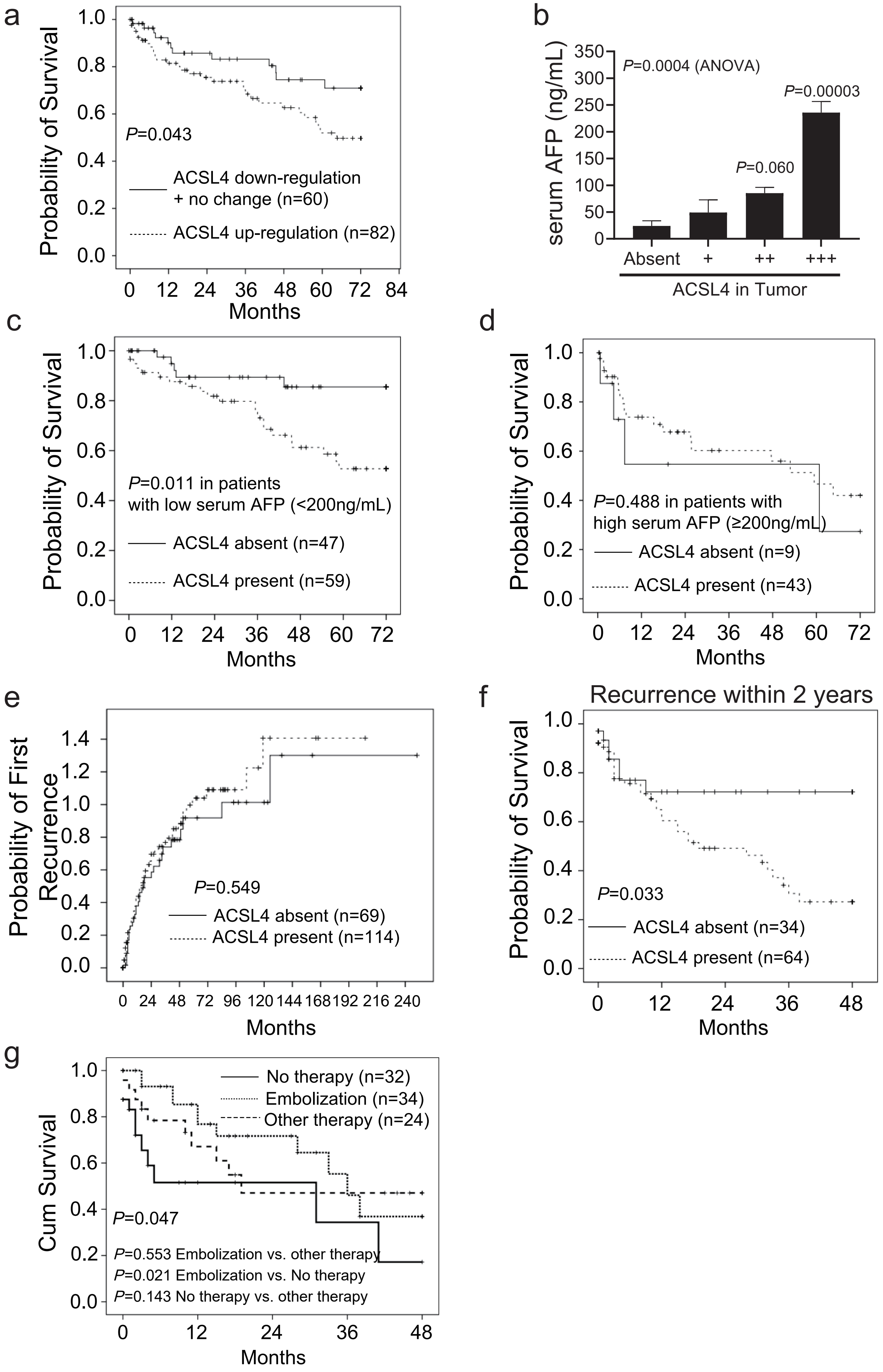

Supplementary figure 2

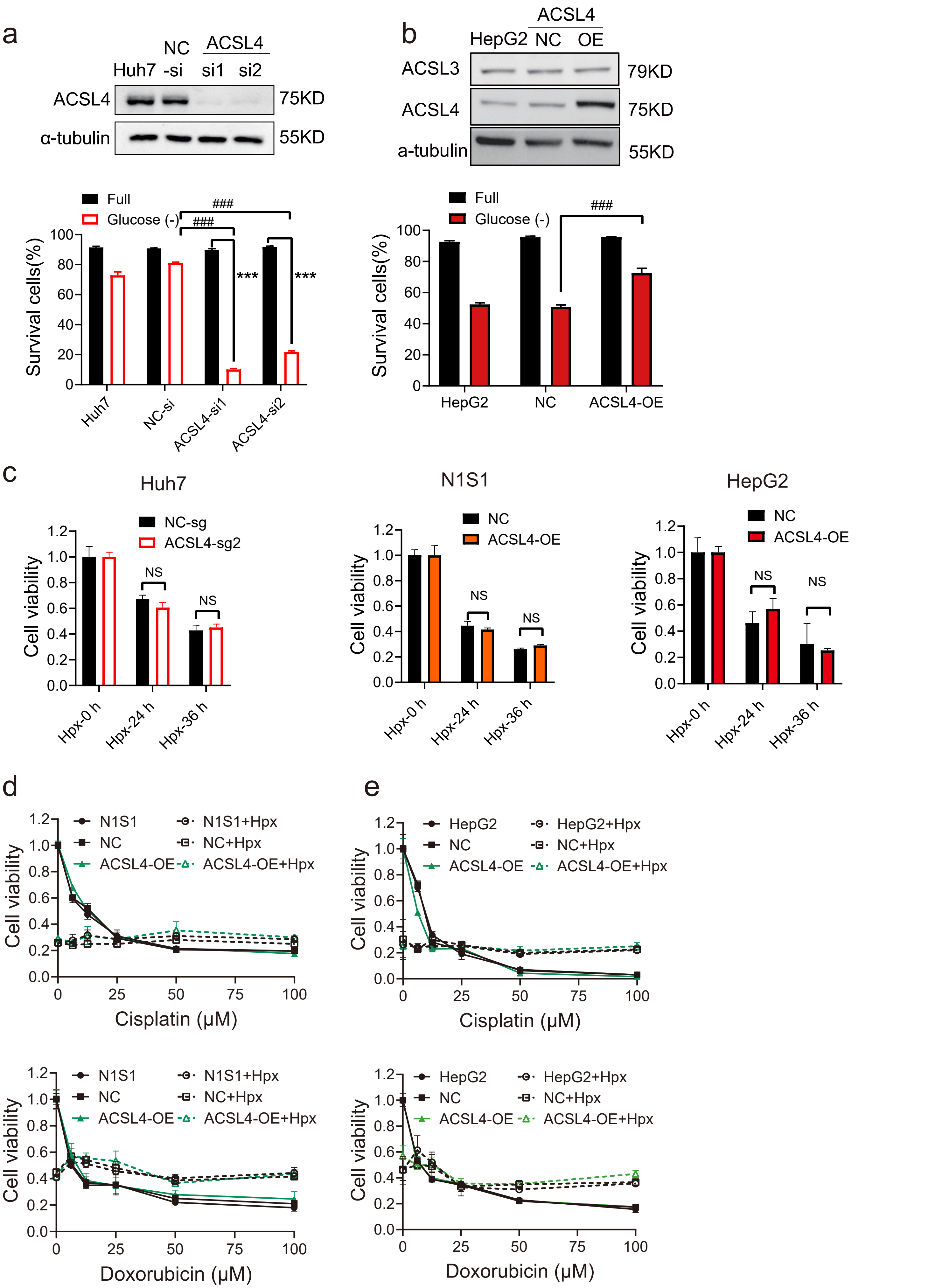

Supplementary figure 3 (a-e)

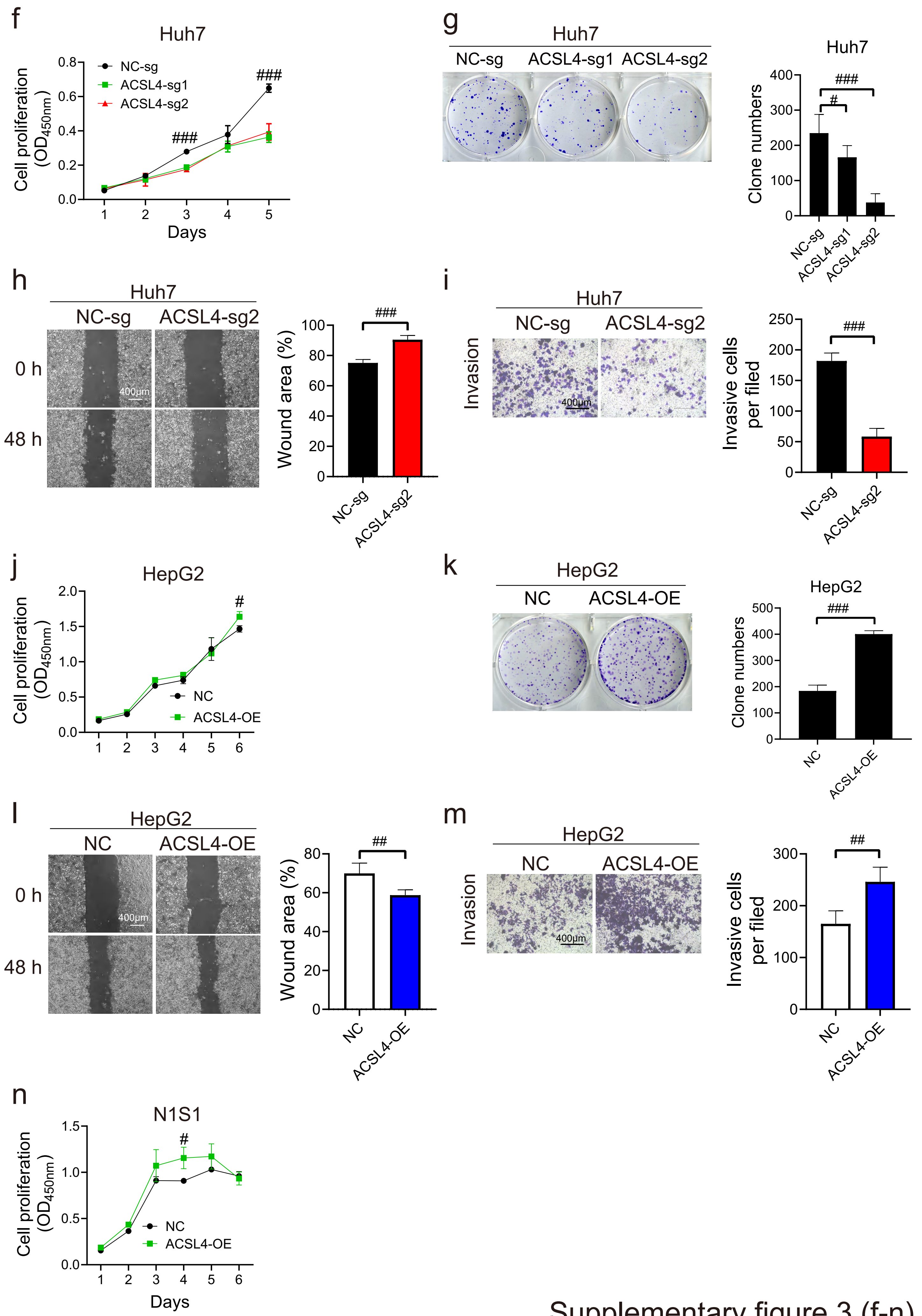

Supplementary figure 3 (f-n)

o

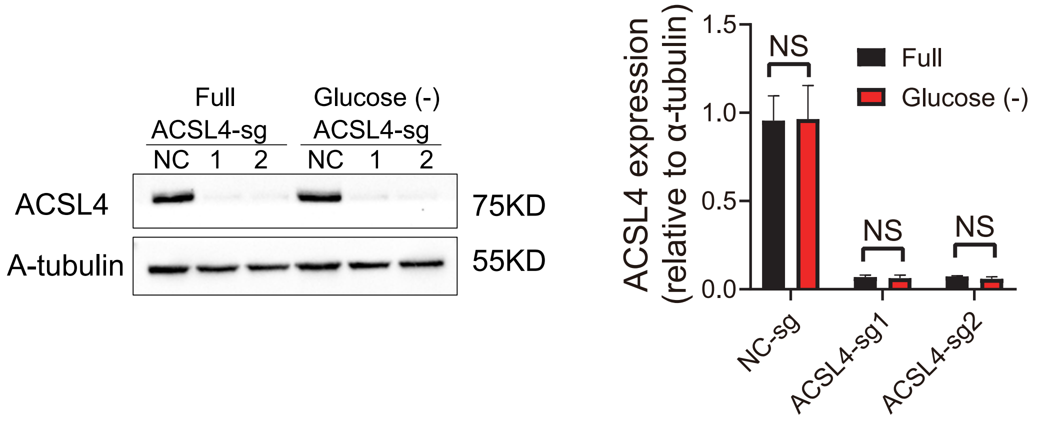

Supplementary figure 3 (o)

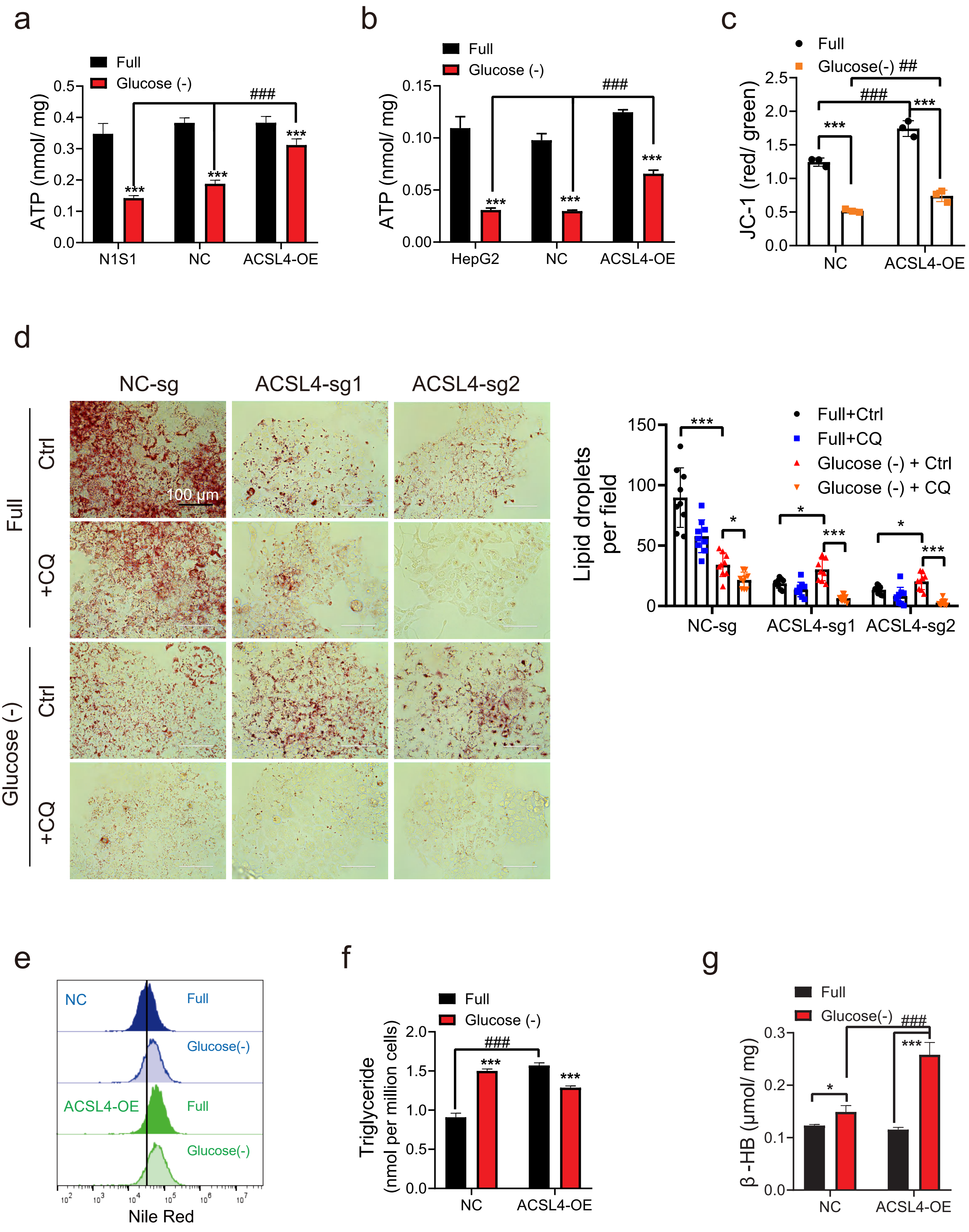

Supplementary figure 4

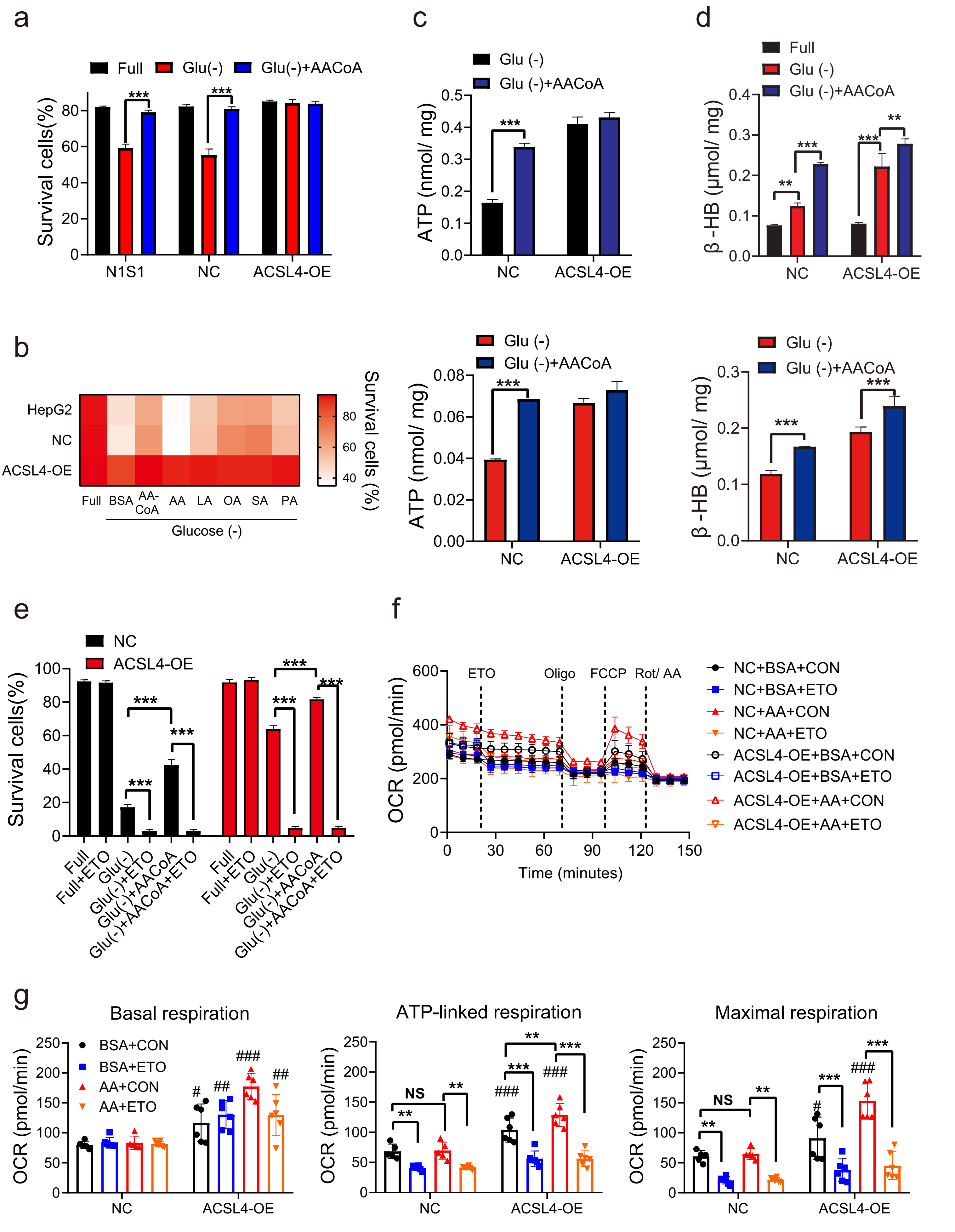

Supplementary figure 5

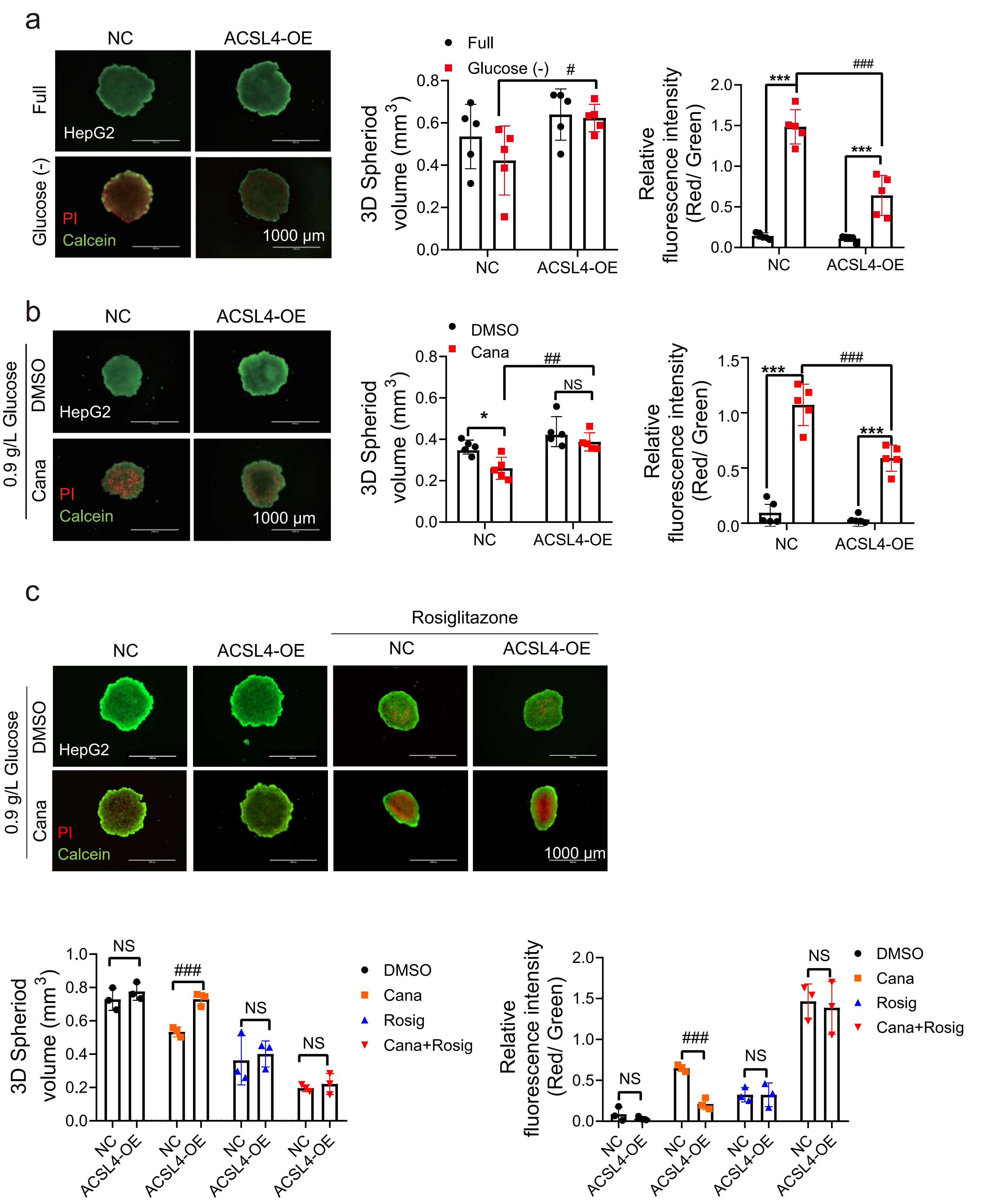

Supplementary figure 6 (a-c)

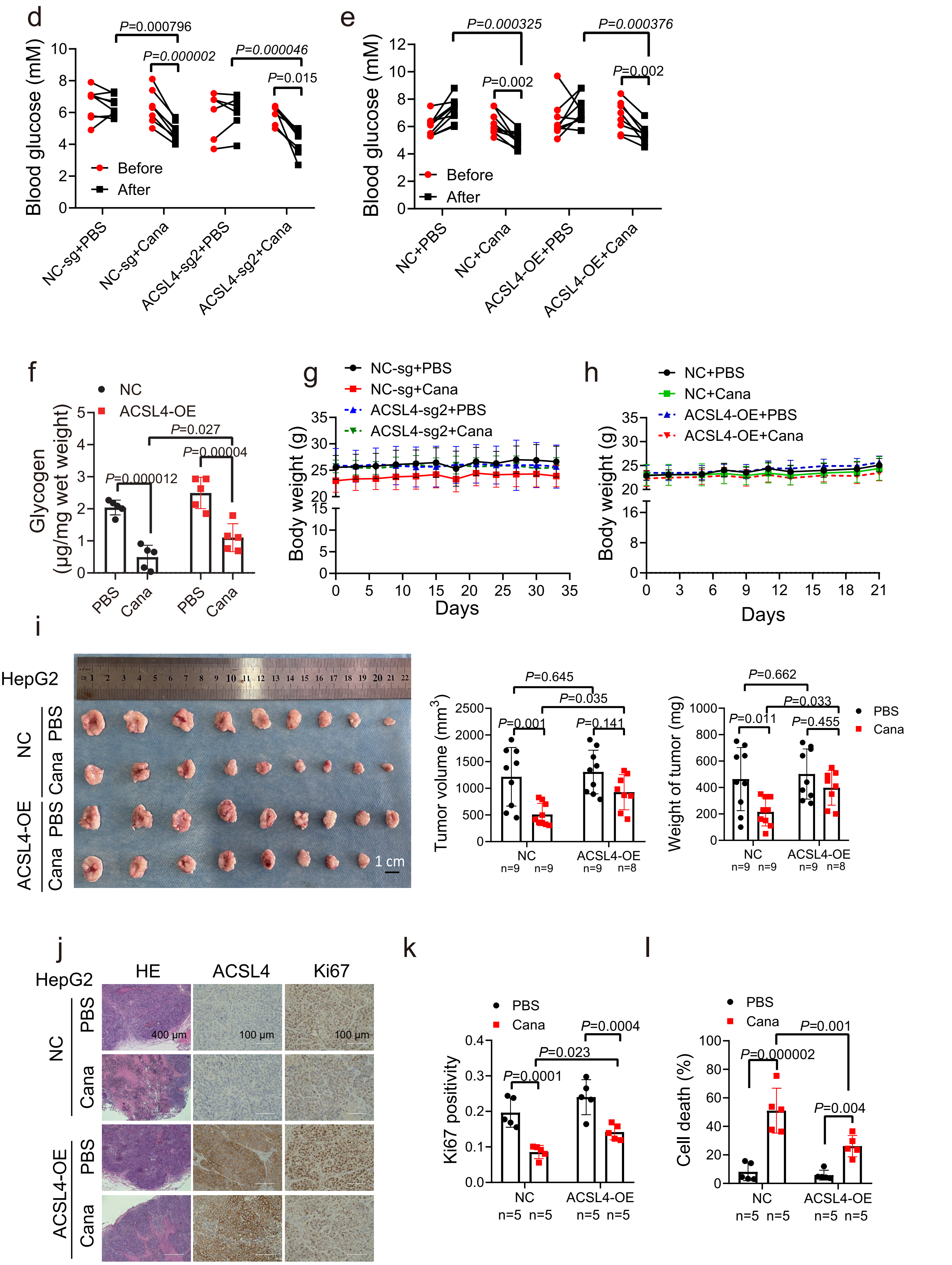

Supplementary figure 6 (d-l)

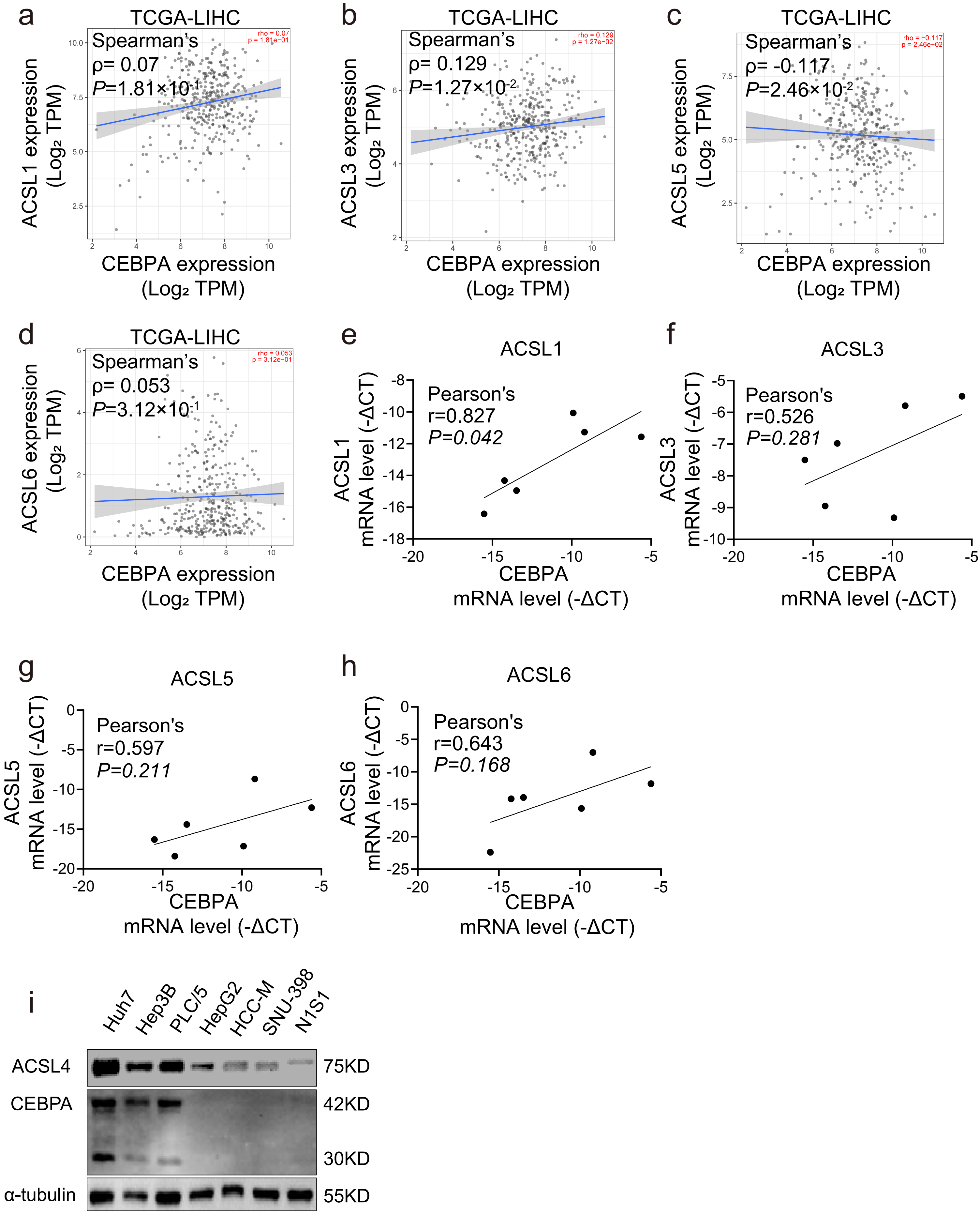

Supplementary figure 7

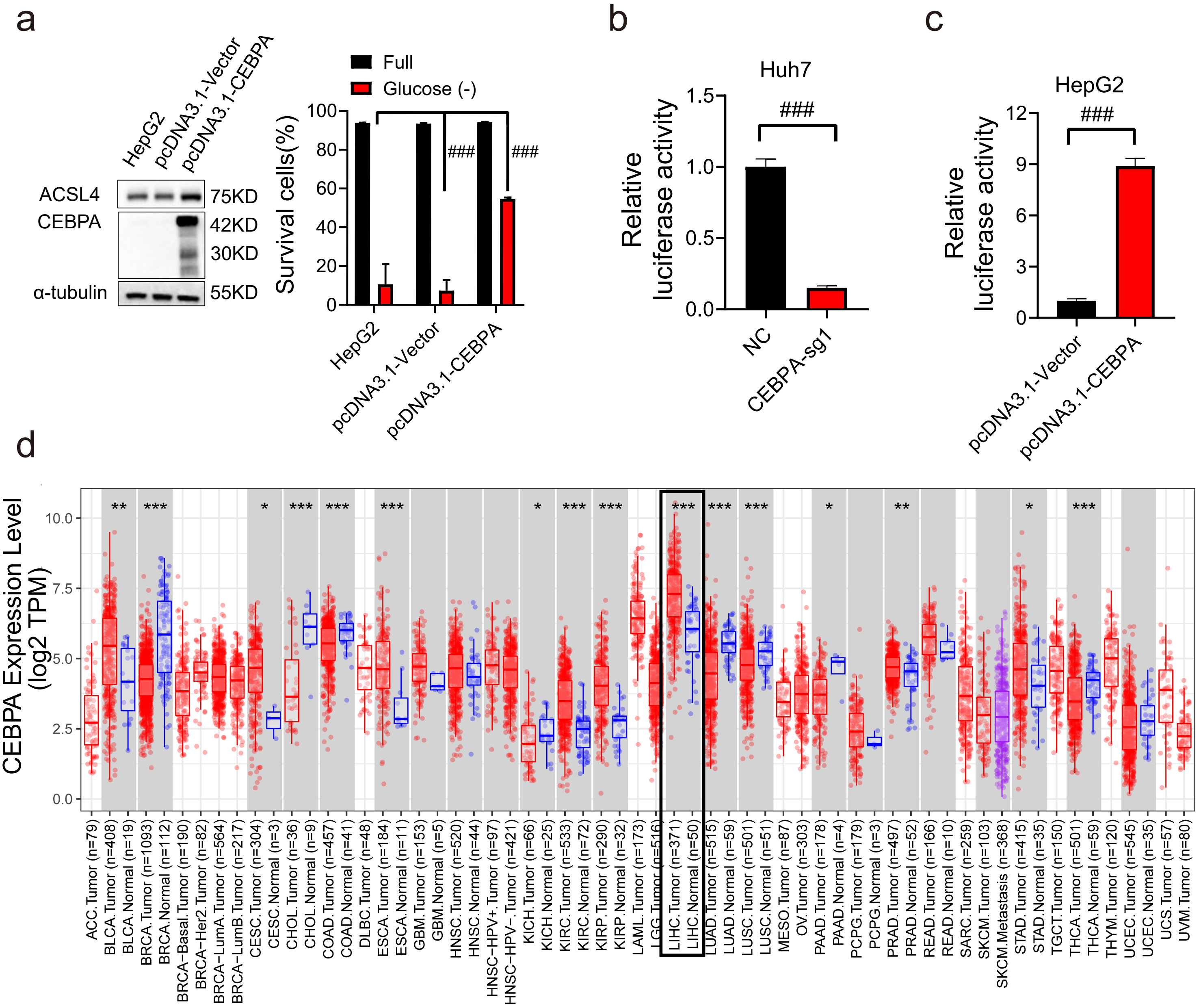

e

ACSL4 expression in HCC	2nd Attack of recurrence after post-recurrent TACE (*P*=0.076)		Total
	Yes	No	
High	4(100%)	0	4
Low	2(33.3%)	4	6
Total	6	4	10

f

CEBPA positivity in HCC	2nd Attack of recurrence after post-recurrent TACE (*P*=0.524)		Total
	Yes	No	
High	4(80%)	1	5
Low	2(40%)	3	5
Total	6	4	10

Supplementary figure 8
