# Supplementary material for: The prognostic role of ACSL4 in postoperative adjuvant TACE-treated HCC: implications for therapeutic response and mechanistic insights
Source: J Exp Clin Cancer Res. 2024 Nov 19;43:306. doi: 10.1186/s13046-024-03222-5 (PMC11575417; doi:10.1186/s13046-024-03222-5)
Supplement: Supplementary file 3 — Supplementary Material 3. [file 13046_2024_3222_MOESM3_ESM.docx]

**Supplementary Table 1: The common clinicopathological parameters of primary HCC patients with or without ACSL4 expression in tumor (Singapore HCC cohort).**

| **Parameter** | **ACSL4 absent in tumor (N=69)** | **ACSL4 present in tumor (N=114)** | ***P*-**  **value** |
| --- | --- | --- | --- |
| Age (mean ± SD years) | 60.7 ± 10.0 | 56.5 ± 15.1 | **0.026** |
| Gender: Male (%) | 61 (88.4%) | 85 (74.6%) | **0.024** |
| Race: Chinese (%) | 54 (78.3%) | 88 (77.9% in 113 cases) | 1.000 |
| HBV carrier (% in x cases) | 39 (61.9% in 63 cases) | 75 (68.2% in 110 cases) | 0.410 |
| HCV carrier (% in x cases) | 6 (9.2% in 65 cases) | 6 (5.5% in 110 cases) | 0.366 |
| BCLC stage  0  A  B | 1  35 (76.1%)  10 (21.7%) | 1  54 (77.1%)  15 (21.4%) | 0.954 |
| Child-Pugh class  A  B  C | 2  36 (80.0%)  7 (15.6%) | 1  59 (85.5%)  9 (13.0%) | 0.563 |
| Smoking  Non-smoker  Ex-smoker  Smoker | 34 (63.0%)  13  7 | 60 (63.2%)  20  15 | 0.849 |
| Drinking  Non-drinker  Ex- drinker  Drinker | 30 (54.5%)  10  15 | 65 (66.3%)  16  17 | 0.284 |
| Diabetes mellitus (%) | 24 (36.4% in 66 cases) | 22 (19.8% in 111 cases) | **0.015** |
| Hypertension (%) | 26 (40.0% in 65 cases) | 22 (31.5% in 111 cases) | 0.325 |
| Fatty liver (%) | 7 (10.1%) | 17 (14.9%) | 0.498 |
| Liver fibrosis | 8 (11.6%) | 28 (24.6%) | **0.036** |
| Cirrhosis (%) | 39 (56.5%) | 59 (51.8%) | 0.545 |
| Tumor size (%):  < 2 cm  2~ 5 cm  ≥ 5 cm | 6 (8.8%)  28 (41.2%)  34 (50.0%) | 13 (11.6%)  49 (43.8%)  50 (44.6%) | 0.727 |
| Multiple tumor nodules (%) | 23 (33.3%) | 32 (28.7%) | 0.559 |
| Tumor stage (%):  I  II  III & IV | 32 (50.0%)  21 (32.8%)  11 (17.2%) | 54 (49.1%)  31 (28.2%)  25 (22.7%) | 0.639 |
| Serum AFP level  (mean ± SD, ng/mL) | 24.9 ± 9.7 | 112.9 ± 20.3 | **0.001** |
| Vascular invasion (%) | 17 (30.4% in 56 cases) | 39 (40.2% in 97 cases) | 0.296 |
| Tumor necrosis (%) | 43 (62.3%) | 48 (42.5% in 113 cases) | **0.007** |
| Recurrence within 2 years (%) | 22 (31.9%) | 48 (42.1%) | 0.209 |

**Supplementary Table 2: The common clinicopathological parameters of ACSL4-high or -low HCC patients who had post-operative adjuvant TACE/TAE treatment (Guangxi HCC cohort).**

| **Parameter** | **ACSL4 expression in HCC** | | ***P*-value** |
| --- | --- | --- | --- |
|  | **High (N=36)** | **Low (N=34)** |  |
| Age (mean ± SD) | 49.00 ± 10.75 | 50.65 ± 9.52 | 0.289 |
| Gender: Male (%) | 34 (94.4%) | 31 (91.2%) | 0.673 |
| HBV carrier (%) | 24 (66.6%) | 24 (70.6%) | 0.724 |
| HCV carrier (%) | 0 | 1 (2.9%) | 1 |
| BCLC stage |  |  | 0.549 |
| 0-A | 27 | 27 |  |
| B | 5(13.9%) | 2 (5.9%) |  |
| C | 4(11.1%) | 5(14.7%) |  |
| Child-Pugh class |  |  | NA |
| A | 35 | 34 |  |
| B | 1(2.8%) | 0 |  |
| CNLC stage |  |  | 0.756 |
| Ⅰa-Ⅱa | 22 (61.1%) | 22 (64.7%) |  |
| Ⅱb-Ⅲa | 14 | 12 |  |
| Smoking |  |  | 0.054 |
| Non-smoker | 27 (75%) | 18 (52.9%) |  |
| Smoker | 9 | 16 |  |
| Drinking |  |  | 0.157 |
| Non-drinker | 13 (36.1%) | 18 (52.9%) |  |
| Drinker | 23 | 16 |  |
| Diabetes mellitus (%) | 1 (2.8%) | 1 (2.9%) | 1 |
| Hypertension (%) | 4 (11.1%) | 5 (14.7%) | 0.927 |
| Fatty liver (%) | 1 (2.8%) | 3 (8.8%) | 0.567 |
| Cirrhosis (%) | 22 (61.6%) | 13 (38.2%) | **0.056** |
| Microvascular invasion |  |  |  |
| M0 | 36 | 36 | NA |
| M1 or M2 | 0 | 0 |  |
| Portal vein tumor thrombus (%) | 12 (33.3%) | 12 (35.3%) | 0.863 |
| Tumor size (%) |  |  | 0.204 |
| < 2 cm | 5 (13.9%) | 1 (2.9%) |  |
| 2~5 cm | 17 (47.2%) | 16 (47.1%) |  |
| ≥5 cm | 14 (38.9%) | 17 (50%) |  |
| Multiple tumor nodules (%) | 7 (19.4%) | 5 (14.7%) | 0.599 |
| AFP (ng/mL) |  |  |  |
| Before Hepatectomy |  |  | 0.075 |
| ≥ 200 | 6 (16.7%) | 12 (35.3%) |  |
| After Hepatectomy |  |  | 0.282 |
| ≥ 200 | 9 (25%) | 5 (14.7%) |  |

Continued to **Supplementary Table 2**

| **Parameter** | **ACSL4 expression in HCC** | | ***P*-value** |
| --- | --- | --- | --- |
|  | **High (N=36)** | **Low (N=34)** |  |
| AFP (ng/mL) |  |  |  |
| Before post-recurrent TACE |  |  | 0.807 |
| ≥ 200 | 6 (16.7%) | 4 (11.8%) |  |
| After post-recurrent TACE |  |  | 0.073 |
| ≥ 200 | 9 (25%) | 3 (8.8%) |  |
| Tumor necrosis (%) | 4 (11.1%) | 13 (38.2%) | **0.008** |

**Supplementary Table 3: The common clinicopathological parameters of ACSL4-high or -low recurrent HCC patients who had post-recurrence TACE/TAE treatment (Guangxi-HCC cohort).**

| **Parameter** | **ACSL4 expression in HCC** | | ***P*-value** |
| --- | --- | --- | --- |
|  | **High (N=4)** | **Low (N=6)** |  |
| Age (mean ± SD) | 56.00±6.98 | 49.00±10.81 | 0.289 |
| Gender: Male (%) | 4 (100%) | 5 (83.3%) | 0.673 |
| HBV carrier (%) | 2 (50%) | 5 (83.3%) | 0.400 |
| HCV carrier (%) | 1 | 0 | 1 |
| BCLC stage |  |  | 0.549 |
| 0-A | 4 | 3 |  |
| B | 0 | 1 (16.7%) |  |
| C | 0 | 2 (33.3%) |  |
| Child-Pugh class |  |  | NA |
| A | 4 | 6 |  |
| B | 0 | 0 |  |
| CNLC stage |  |  | 0.571 |
| Ⅰa-Ⅱa | 3 (75%) | 3 (50%) |  |
| Ⅱb-Ⅲa | 1 | 3 |  |
| Smoking |  |  | 0.5 |
| Non-smoker | 2 (50%) | 5 (83.3%) |  |
| Smoker | 2 | 1 |  |
| Drinking |  |  | 1 |
| Non-drinker | 2 (50%) | 4 (66.6%) |  |
| Drinker | 2 | 2 |  |
| Diabetes mellitus (%) | 1 (25%) | 0 | 0.4 |
| Hypertension (%) | 1 (25%) | 0 | 0.4 |
| Fatty liver (%) | 1 (25%) | 3 (50%) | 0.571 |
| Cirrhosis (%) | 4 (100%) | 1 (16.7%) | **0.048** |
| Microvascular invasion |  |  |  |
| M0 | 4 | 6 | NA |
| M1 or M2 | 0 | 0 |  |
| Portal vein tumor thrombus (%) | 1 (25 %) | 3 (50%) | 0.571 |
| Tumor size (%) |  |  | 0.5 |
| <2 cm | 0 | 0 |  |
| 2~5 cm | 2 (50%) | 1 (16.7%) |  |
| ≥5 cm | 2 (50%) | 5 (83.3%) |  |
| Multiple tumor nodules (%) | 1 (25%) | 3 (50%) | 0.571 |
| AFP (ng/mL) |  |  |  |
| Before Hepatectomy |  |  | 0.133 |
| ≥ 200 | 2 (50%) | 6 (100%) |  |
| After Hepatectomy |  |  | 1 |
| ≥ 200 | 3 (75%) | 4 (66.7%) |  |

Continued to **Supplementary Table 3**

| **Parameter** | **ACSL4 expression in HCC** | | ***P*-value** |
| --- | --- | --- | --- |
|  | High (N=4) | Low (N=6) |  |
| AFP (ng/mL) |  |  |  |
| Before TACE |  |  | 1 |
| ≥ 200 | 3 (75%) | 4 (66.7%) |  |
| After TACE |  |  | 1 |
| ≥ 200 | 3 (75%) | 5 (83.3%) |  |
| Tumor necrosis (%) | 0 | 2 (33.3%) | 0.467 |
